# Impaired antibacterial autophagy links granulomatous intestinal inflammation in Niemann–Pick disease type C1 and XIAP deficiency with NOD2 variants in Crohn's disease

**DOI:** 10.1136/gutjnl-2015-310382

**Published:** 2016-03-07

**Authors:** Tobias Schwerd, Sumeet Pandey, Huei-Ting Yang, Katrin Bagola, Elisabeth Jameson, Jonathan Jung, Robin H Lachmann, Neil Shah, Smita Y Patel, Claire Booth, Heiko Runz, Gesche Düker, Ruth Bettels, Marianne Rohrbach, Subra Kugathasan, Helen Chapel, Satish Keshav, Abdul Elkadri, Nick Platt, Alexio M Muise, Sibylle Koletzko, Ramnik J Xavier, Thorsten Marquardt, Fiona Powrie, James E Wraith, Mads Gyrd-Hansen, Frances M Platt, Holm H Uhlig

**Affiliations:** 1 Translational Gastroenterology Unit, University of Oxford, Oxford, UK; 2 Nuffield Department of Clinical Medicine, Ludwig Institute for Cancer Research, University of Oxford, Oxford, UK; 3 Willink Biochemical Genetics Unit, Manchester Centre for Genomic Medicine, Saint Mary's Hospital, Manchester, UK; 4 National Hospital for Neurology and Neurosurgery, London, UK; 5 Great Ormond Street Hospital, London, UK; 6 NIHR Oxford Biomedical Research Centre, University of Oxford, Oxford, UK; 7 Department of Clinical Immunology, Great Ormond Street Hospital, London, UK; 8 University of Heidelberg, Heidelberg, Germany; 9 University Children's Hospital Bonn, Bonn, Germany; 10 Children's Hospital Münster, Münster, Germany; 11 Children's Research Centre Zurich, University Children's Hospital, Zurich, Switzerland; 12 Division of Pediatric Gastroenterology, Emory University School of Medicine, Atlanta, Georgia, USA; 13 SickKids Inflammatory Bowel Disease Center and Cell Biology Program, Research Institute, The Hospital for Sick Children, Toronto, Ontario, Canada; 14 Division of Gastroenterology, Hepatology, and Nutrition, Department of Pediatrics, The Hospital for Sick Children, University of Toronto, Toronto, Ontario, Canada; 15 Department of Pharmacology, University of Oxford, Oxford, UK; 16 Dr. von Hauner Children's Hospital, Ludwig-Maximilians-University, Munich, Germany; 17 Broad Institute of Harvard and Massachusetts Institute of Technology, Cambridge, Massachusetts, USA; 18 Kennedy Institute of Rheumatology, University of Oxford, Oxford, UK; 19 Department of Pediatrics, University of Oxford, Oxford, UK

**Keywords:** IMMUNODEFICIENCY, IBD BASIC RESEARCH, IBD CLINICAL, IBD - GENETICS, CROHN'S DISEASE

## Abstract

**Objective:**

Patients with Niemann–Pick disease type C1 (NPC1), a lysosomal lipid storage disorder that causes neurodegeneration and liver damage, can present with IBD, but neither the significance nor the functional mechanism of this association is clear. We studied bacterial handling and antibacterial autophagy in patients with NPC1.

**Design:**

We characterised intestinal inflammation in 14 patients with NPC1 who developed IBD. We investigated bacterial handling and cytokine production of NPC1 monocytes or macrophages in vitro and compared NPC1-associated functional defects to those caused by IBD-associated nucleotide-binding oligomerization domain-containing protein 2 (NOD2) variants or mutations in X-linked inhibitor of apoptosis (XIAP).

**Results:**

Patients with the lysosomal lipid storage disorder NPC1 have increased susceptibility to early-onset fistulising colitis with granuloma formation, reminiscent of Crohn's disease (CD). Mutations in NPC1 cause impaired autophagy due to defective autophagosome function that abolishes NOD2-mediated bacterial handling in vitro similar to variants in NOD2 or XIAP deficiency. In contrast to genetic NOD2 and XIAP variants, NPC1 mutations do not impair NOD2-receptor-interacting kinase 2 (RIPK2)-XIAP-dependent cytokine production. Pharmacological activation of autophagy can rescue bacterial clearance in macrophages in vitro by increasing the autophagic flux and bypassing defects in NPC1.

**Conclusions:**

NPC1 confers increased risk of early-onset severe CD. Our data support the concept that genetic defects at different checkpoints of selective autophagy cause a shared outcome of CD-like immunopathology linking monogenic and polygenic forms of IBD. Muramyl dipeptide-driven cytokine responses and antibacterial autophagy induction are parallel and independent signalling cascades downstream of the NOD2-RIPK2-XIAP complex.

**Significance of this study**

**What is already known on this subject?**

- Defects in host–bacterial interactions cause IBD.
- Defects in autophagy are associated with delayed elimination of intracellular bacteria and intestinal inflammation, as observed in Crohn's disease.
- Monogenic forms of IBD inform about non-redundant pathways in the mucosal immune system.

**What are the new findings?**

- Mutations in Niemann–Pick disease type C1 (NPC1) predispose to early-onset IBD with Crohn's disease phenotype and granuloma formation.
- NPC1 defects impair the elimination of intracellular bacteria in macrophages due to dysfunctional autophagy.
- NPC1-associated intestinal inflammation shares a functional defect of impaired antibacterial autophagy with Crohn's disease-associated variants in nucleotide-binding oligomerization domain-containing protein 2 and mutations in X-linked inhibitor of apoptosis, a cause of monogenic IBD, but muramyl dipeptide-initiated cytokine production is not affected in patients with NPC1.
- Rescue of lysosomal lipid storage defect in NPC1 macrophages by US Food and Drug Administration-approved cyclodextrin does not restore antibacterial autophagy, whereas autophagy induction by chlorpromazine can rescue the impaired bacterial handling phenotype.

**How might it impact on clinical practice in the foreseeable future?**

- Patients with NPC1 and GI problems should be screened for Crohn's disease-like intestinal inflammation.
- Induction of autophagy is a potential therapeutic strategy to overcome genetic autophagy defects.

## Introduction

Niemann–Pick type C (NPC) is a neurodegenerative lysosomal storage disorder^1^ associated with defects in lysosomal calcium homeostasis and lipid trafficking.^2^ It is caused by mutations in the *NPC1* or *NPC2* genes.^1^ The accumulation of unesterified cholesterol and multiple sphingolipids in the late endosomal/lysosomal system causes neurological and visceral symptoms. So far, no specific immune dysfunction has been linked to NPC. However, a high proportion of patients develop airway infections.^3^ Two cases of Crohn's-like disease were reported in patients with genetically confirmed NPC1 mutations.^4^ ^5^

IBD is a multifactorial disorder with genetic susceptibility, immunological predisposition and environmental triggers.^6^ ^7^ To date, >150 genetic loci have been linked to IBD by association studies.^8^ Variants in genes that affect bacterial handling (such as nucleotide-binding oligomerization domain-containing protein 2 NOD2) and autophagy (such as ATG16L1 or IRGM) are associated with polygenic IBD. Variants in NOD2 are the strongest genetic risk factor for Crohn's disease (CD).^7^ ^9^ ^10^ NOD2 plays a key role in bacterial handling in dendritic cells and in the epithelium.^11–13^

In addition to polygenic IBD comprising CD and ulcerative colitis (UC), there are an increasing number of monogenic disorders presenting with IBD and highlighting a role of bacterial handling in innate immune cells.^14–16^ In male patients, mutations in the gene encoding X-linked inhibitor of apoptosis (XIAP) cause an immune-dysregulation syndrome characterised by haemophagocytic lymphohistiocytosis and further inflammatory complications. Notably, one-fifth of patients with XIAP deficiency develop severe CD-like granulomatous colitis.^17–20^ More recently, the ubiquitin (Ub) ligase XIAP emerged as an essential signal transducer downstream of the cytosolic sensor NOD2.^21^ ^22^ Following activation of NOD2 by muramyl dipeptide (MDP), a bacterial cell wall product, XIAP binds and ubiquitinates the adapter protein receptor-interacting kinase 2 (RIPK2) to facilitate nuclear factor (NF)-κB signalling and cytokine production.^23^ Multiple Ub-dependent signalling events regulate NOD2 activity and converge on the adapter protein RIPK2.^24^ ^25^

Here, we report that antibacterial autophagy initiated by the NOD2-RIPK2-XIAP pathway is a key defect in disorders presenting with granulomatous intestinal inflammation and this defect can be independent of MDP-mediated cytokine production. Based on a case series of 14 patients with NPC1 mutations who developed early-onset CD-like disease with granuloma formation and patients with mutations in NOD2 and XIAP, we provide evidence of a shared defect of degradation of bacteria, such as *Salmonella enterica serovar typhimurium* (*S. typhimurium*) and CD-associated adherent-invasive *Escherichia coli* (AIEC). In contrast to patients with NOD2 and XIAP variants, MDP-induced cytokine secretion via NOD2 and XIAP is intact in patients with NPC1. Our results suggest that in NPC1 autophagosomal maturation rather than lysosomal dysfunction affects autophagic elimination of intracellular bacteria. In our model, dysregulated cytokine response is the consequence of incomplete bacterial clearance and pharmaceutical induction of autophagy can restore bacterial killing, suggesting a potential therapeutic strategy.

## Materials and methods

### Research subjects

Participating centres contributed anonymised patient data or blood samples with local ethics. Historic patient notes were only reviewed if written informed consent for research was available. Adult patients with NPC were able to give full informed consent. Healthy control blood samples were obtained from healthy volunteer donors (healthy control group I) or as leucocyte cones (healthy control group II) from UK blood donor bank.

#### Patients with NPC1

NPC1-IBD case-finding was performed in several European and US centres, specialised in NPC patient care. In addition, searching the ehealthMe database (http://www.ehealthme.com) for NPC and IBD/colitis/CD revealed six patient reports (range 2–19 years) that allowed retrieval of adverse outcome reports of the Center of Disease Control (Atlanta, USA) reporting IBD-like immunopathology in patients with NPC1 who had received miglustat treatment. Phenotype data were captured using a structured survey. For functional experiments, blood samples were obtained from six patients with NPC1 (two female, median age 29 years, range 13–50 years, three patients on miglustat). Among those, four patients did not have IBD. Further details are provided in online supplementary materials.

10.1136/gutjnl-2015-310382.supp1 Supplementary data

#### NOD2 and XIAP patients

Twenty-six IBD patients with wild-type (WT) or variant NOD2 genotype were recruited from the Oxford IBD cohort study, which is a single-centre prospective cohort study investigating patients with IBD across all ages (Oxford IBD cohort study, manuscript in preparation). Carriers of NOD2 polymorphisms were identified by Immunochip. Eight individuals with genetically confirmed XIAP mutations were recruited from centres for immunodeficiency in Oxford and London, UK, and Toronto, Canada. For patient details, see online supplementary tables.

### Differentiation and culture of cells

Peripheral blood mononuclear cells (PBMC) were isolated by Ficoll gradient centrifugation. Monocyte-derived macrophages (MDM) were differentiated from the adherent fraction of PBMC over 5 days in RPMI-1640 medium supplemented with 10% fetal calf serum (Sigma-Aldrich) and 100 ng/mL macrophage colony-stimulating factor (M-CSF) (R&D Systems).

### Stimuli and cytokines

PBMC or MDM were stimulated with MDP (10 µg/mL), lipidated L18-MDP (200 ng/mL), inactive D-D isomer of MDP (10 µg/mL), Flagellin from *S. typhimurium* (100 ng/mL), Pam3CSK4 (100 ng/mL, all Invivogen), Lipopolysaccharide (LPS) from *Salmonella minnesota* R595 (20–200 ng/mL, Enzo), interleukin (IL)-10 (20 ng/mL), interferon (IFN)-γ (50 ng/mL) or tumour necrosis factor (TNF) (10 ng/mL, all Peprotech). Lipidation of MDP (L18-MDP) allows reduction of MDP concentration in stimulation studies.

### Chemical compounds

Small-molecule inhibitors were added to cell culture medium 1 h prior to stimulation. ML-130 (5 µM) and ponatinib (50 nM) were purchased from Selleck Chemical. Compound 21a (XIAP-Cp-21a, 1–2.5 µM) was kindly provided by TetraLogics Pharmaceuticals.^26–28^ To induce the lysosomal lipid storage phenotype in MDM/PBMC, cells were pre-incubated with 2 µg/mL U18666A (Sigma-Aldrich) for 48/24 h. Other compounds were bafilomycin A1 (50 nM, Enzo Life Sciences), 2-hydroxypropyl-β-cyclodextrin (0.5–2%, Sigma-Aldrich), chlorpromazine (1–10 μg/mL, Merck Millipore), rapamycin (1–10 µM, Cayman Chemical), Torin 1 (10 µM, Tocris), d-(+)-trehalose dihydrate (100 mM, Sigma-Aldrich) and miglustat (4–400 µM, Tocris).

### Intracellular flow cytometry

Measurement and quantification of intracellular TNF in monocytes was performed as described previously^29^ and as detailed in online supplementary methods.

### Gentamicin protection assay

Bacterial uptake, intracellular survival and replication were tested with the following strains: *Salmonella enterica serovar typhimurium* (*S. typhimurium*)-expressing green-fluorescent protein (GFP) (NCTC 12023) and CD-associated AIEC reference strain LF82.^30^ Bacteria were freshly grown and used at mid-log phase. MDM in 96-well flat bottom plates were pretreated with inhibitors and subsequently stimulated for 2 h before infection at a multiplicity of infection of 10 bacteria per cell. One hour post infection, new complete medium supplemented with 100 µg/mL gentamicin was added. At 3 h post infection, MDM were washed and lysed in 1% Triton X-100 in deionised water to determine the number of colony-forming units (CFU) recovered from the lysed macrophages. For each donor, conditions were tested in three parallel infection experiments and results are normalised to individual mean CFU of unstimulated or vehicle-treated condition. Results per donor are shown.

### Confocal microscopy

See online supplementary methods.

### Purification of endogenous Ub conjugates

Purification of endogenous Ub conjugates was performed with Tandem Ubiquitin Binding Entities (TUBE) as described previously^23^ and as outlined in online supplementary methods.

### Immunoblotting

See online supplementary methods.

### Statistical analysis

Data analysis was performed using GraphPad Prism V.5.00 (GraphPad Software, San Diego, California, USA). Data were compared using two-sided Mann–Whitney U test. Post hoc testing with analysis of variance and post-test correction with Bonferroni (comparisons of MDP-mediated effects) or Dunnett (multiple comparisons to one single control) confirmed presented statistical significance. Categorical data were compared using two-sided Fisher's exact test and confidence intervals calculated according to the Wald equation. p<0.05 was considered significant (*p<0.05, **p<0.01, ***p<0.001).

## Results

### Patients with symptomatic NPC1 develop early-onset CD-like intestinal inflammation with granuloma

**Figure 1:**
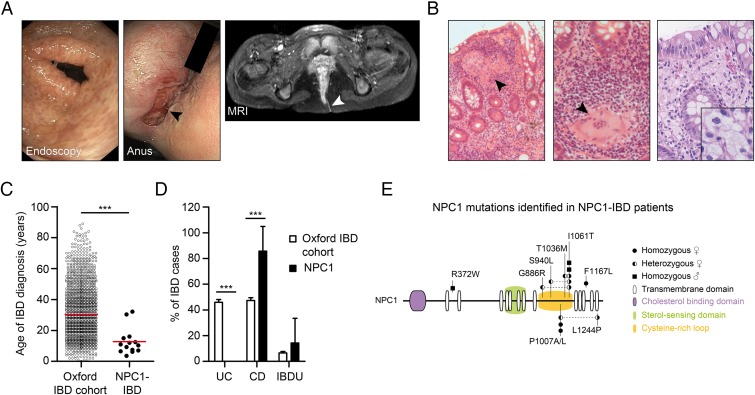
Niemann–Pick disease type C1 (NPC1) is associated with early-onset Crohn's-like immunopathology. (A) Evidence of multiple aphthous lesions on colonoscopy and severe perianal disease complicated by fistulas (arrow head). (B) Colonic biopsies of patients with NPC1-IBD showing large granulomas, polymorphic cell infiltrate (left images, H&E stain) and foam cell macrophages (right image, Periodic acid–Schiff (PAS) stain). (C) Age at IBD diagnosis comparing patients with NPC1-IBD (n=14) with Oxford IBD cohort study (n=2113). (D) Frequency of IBD diagnosis made in the two cohorts. Fisher's exact test, ***p<0.001. (E) NPC1 schematic showing mutations identified in NPC1-IBD cohort. Circles: female patients; squares: male patients.

We studied a cohort of 14 patients with defects in NPC1 who presented with severe CD-like intestinal inflammation and multinucleated granuloma on histopathology (figure 1A, B, table 1 and online supplementary table S1). Granuloma were present in 7 of the 14 patients and foam cell macrophages, indicating lipid storage were present in some (figure 1B).

**Table 1:** IBD phenotype in patients with NPC

Patient ID	Sex	Age at diagnosis of IBD in years	Diagnosis	Symptoms of IBD/EIM/examination findings	Disease location, disease behaviour, growth delay*	Treatment for IBD: enteral nutrition, anti-inflammatory/immunosuppressive drugs	Surgery
1	F	9.3	CD	Diarrhoea, abdominal pain, rectal bleeding, weight loss, perianal skin tags, arthritis	L2L4a, B1p G1	CS, AB, ASA, AZA, IFX (6-weekly)	None
2	M	32.2	IBDU	Diarrhoea, rectal bleeding, weight loss	No details	ASA	None
3	F	11	CD	Diarrhoea, pain on defecation, fever, labial abscess, perianal skin tags	L2, B1p	CS, AB, ASA, AZA†	None
4	F	6.2	CD	Diarrhoea, perianal skin tags and fissure	L2, B1p	No therapy at present	None
5	F	10.3	CD	Diarrhoea, anogenital ulcers, perianal skin tags and fissures	L3L4ab, B1p	CS, ASA, AZA, IFX†	None
6	F	7.5	CD	Diarrhoea, rectal bleeding, pain on defecation, perianal skin tags and fissures, perianal and rectovaginal fistulae; later, cutaneous fistulae and abscesses around stomas	L2, B3p	CS, AB, NUT, ASA, IFX†	End colostomy with distal mucus fistula (8 years)
7	F	6.5	CD	Diarrhoea, perianal skin tags	Perianal disease	No therapy at present	None
8	F	12.4	CD	Diarrhoea, rectal bleeding, pain on defecation, perianal fissures, anorectal fistula, arthritis	L2, B1p	CS, AB, NUT, ASA, IFX	Operation of fissures
9	M	3.6	IBDU	Diarrhoea, iron deficiency anaemia	E4 G1	NUT, AZA	None
10	F	7	CD	Diarrhoea, pain on defecation, perianal skin tags and fistula	Perianal disease		
11	F	15	CD	Diarrhoea, abdominal pain, rectal bleeding, weight loss, perianal skin tags and fistulae, arthritis, skin lesions	L2, B1p G1	CS, AB, NUT, AZA, IFX	Colectomy and ileostomy (16 years)
12	M	30.4	CD	Diarrhoea, skin lesions	L2, B1	CS, ASA, IFX	None

Patients 6 and 10 have been previously reported in the literature.^4^ ^31^ Additional patient information based on literature only is summarised in online supplementary table S1.

*According to Paris classification.^32^

†Medication at time of death.

AB, oral or intravenous antibiotics given for treatment of colitis, bowel decontamination, fistula treatment; ASA, 5-aminosalicylic acid; AZA, azathioprine; CD, Crohn's disease, CS, corticosteroids; EIM, extraintestinal manifestations; F, female; IBDU, IBD unclassified; IFX, infliximab; M, male; NPC, Niemann–Pick disease type C; NUT, polymeric/elemental diet.

In patients with NPC1, the mean age at IBD diagnosis was 12.8±8.6 years (range 3.6–32.2 years), which is significantly earlier compared with an unselected IBD cohort (figure 1C). The diagnosis of NPC1 preceded the onset of IBD in all cases and included typical signs and symptoms consistent with NPC1.^1^ Clinical features of IBD were weight loss, diarrhoea, rectal bleeding, anogenital ulceration, fissures and fistulas (table 1). Perianal disease was noted in 11 of 14 patients. The diagnosis of IBD was confirmed by endoscopy and biopsy in all cases. Eighty-six per cent of patients (12/14) were diagnosed with CD-like and two patients with IBD unclassified, confirming a significant association of NPC with CD compared with an unselected IBD cohort (figure 1D).

Treatment of patients with NPC1 with intestinal inflammation involved steroids, 5-aminosalicylates, azathioprine and exclusive enteral nutrition. Step-up therapy with infliximab was required in six patients (50%); two patients underwent colectomy and stoma formation (table 1). Treatment with miglustat, an European Medicines Agency-approved therapy for NPC1^33^ that reduces lipid storage and delays disease progression, was not associated with onset of IBD-like inflammation (see online supplementary figure S1). The spectrum of genetic defects in NPC1-IBD does not suggest a specific genotype–phenotype association (figure 1E).

### Bacterial killing in macrophages is MDP-dependent

**Figure 2:**
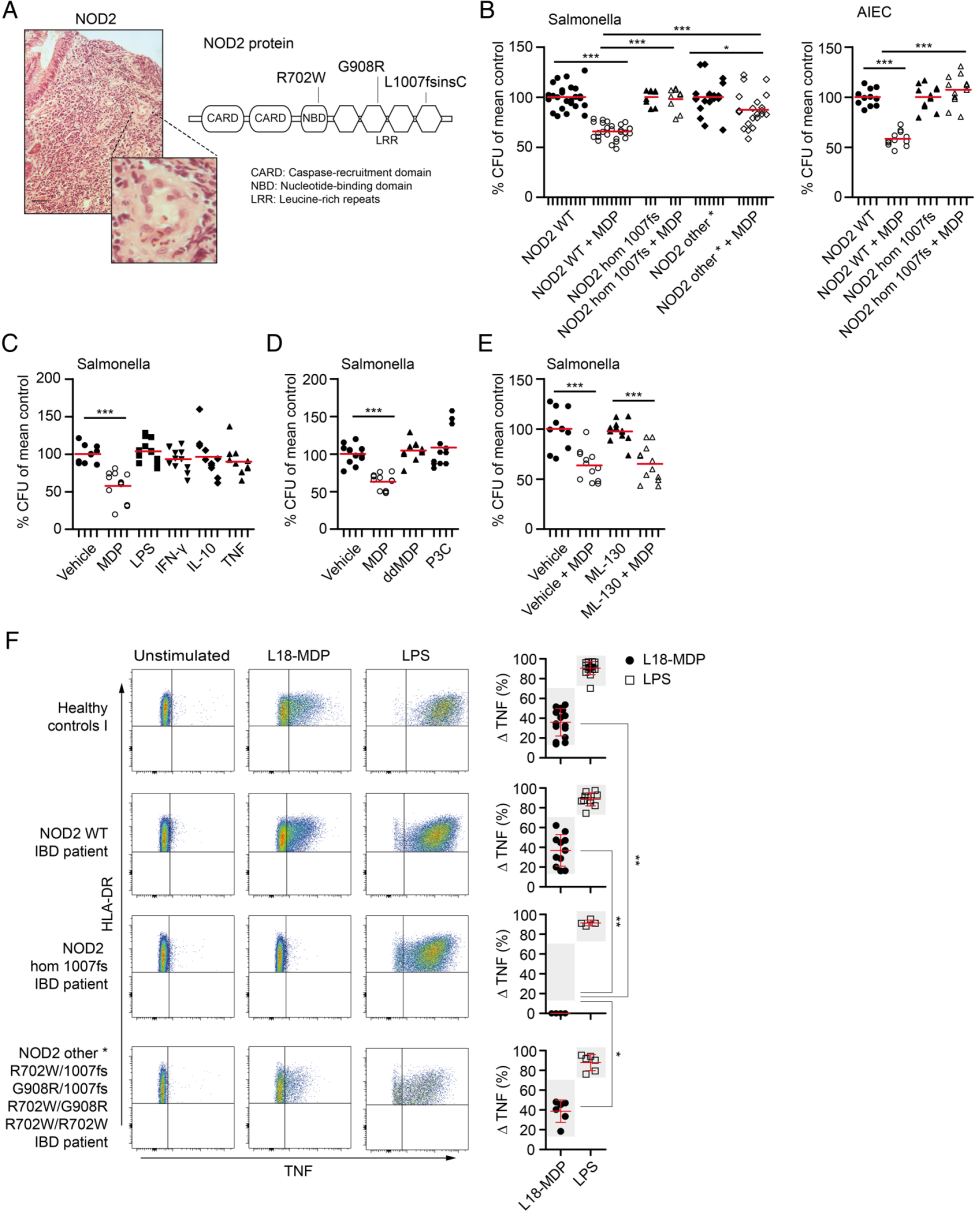
Nucleotide-binding oligomerization domain-containing protein 2 (NOD2) variants impair muramyl dipeptide (MDP)-enhanced bacterial killing in monocyte-derived macrophages (MDM) independently of nuclear factor (NF)-κB signalling. (A) Colonic mucosa of NOD2 patients with Crohn's disease (CD) showing granuloma and inflammation (H&E stain). Enlarged view shows granuloma at higher magnification. Scale bar, 50 μm. NOD2 protein schematic with position of the three most common variants. (B) Gentamicin protection assay with MDM obtained from IBD patients carrying wild-type NOD2 (NOD2 wild-type (WT)), homozygous 1007 fs NOD2 (NOD2 hom 1007 fs) or other homozygous or compound heterozygous combination of NOD2 variants (NOD2 other). Cells were pre-stimulated for 2 h with MDP before exposure to *Salmonella typhimurium* at an multiplicity of infection of 10 for 1 h. Infected MDM were cultured in gentamicin-containing medium for 2 h before cell lysis and quantification of intracellular bacteria. Individual patient results are depicted as indicated by ticks on x-axes (left, n=9 NOD2 WT, n=3 NOD2 hom 1007fs, n=7 NOD2 other) or adherent-invasive *Escherichia coli* (AIEC) (right, n=4 NOD2 WT, n=4 NOD2 hom 1007 fs). For each patient, conditions were tested in three parallel infection experiments and colony-forming units (CFU) were normalised to individual CFU without stimulation. Red bar represents mean. (C) Gentamicin protection assay performed as in (B). MDM were pre-stimulated with MDP, LPS (100 ng/mL), interferon (IFN)-γ, interleukin (IL)-10 or tumour necrosis factor (TNF) and exposed to *S. typhimurium*. Experiments were performed on four healthy controls indicated by ticks on x-axes. Results for individual donors are shown. (D) Bacteria killing assay was performed as in (B) with healthy control MDM (n=4). Following pre-stimulation with MDP, inactive D-D isomer MDP (ddMDP) or Pam3CSK4 (P3C), cells were infected with *S. typhimurium*. (E) Gentamicin protection assay performed as in (B) with healthy control MDM (n=4). Quantification of *S. typhimurium*-CFU after inhibition of NOD1 with ML-130 and MDP pre-stimulation. (F) Representative flow cytometry plots and quantification of TNF-producing HLA-DR^+^CD14^+^ monocytes after stimulation with L18-MDP or LPS (200 ng/mL) in healthy controls (n=16) and IBD patients with wild-type NOD2 (n=11 NOD2 WT), homozygous 1007 fs NOD2 (n=4 NOD2 hom fs1007) or other homozygous or compound heterozygous combination of NOD2 variants (n=6 NOD2 other). Data represent mean±SD. Grey background indicates normal range. p Values were throughout calculated using Mann–Whitney U test. *p<0.05, **p<0.01, ***p<0.001.

We initially assessed whether NPC1 is involved in gene-interaction or protein-interaction networks that include established IBD loci^8^ or known monogenic IBD variants.^15^ We could not predict those interactions (see online supplementary figure S2). Prompted by the finding of large multinucleated granuloma and CD phenotype in patients with NPC1-IBD (figure 1B), we assumed similarities in the pathogenic mechanism. In CD pathogenesis, defects of antibacterial autophagy (a process termed xenophagy) in phagocytes lead to delayed clearance of intestinal microbiota penetrating the epithelial barrier, which might ultimately give rise to granuloma formation (figure 2A).^34^ Interestingly, recently, the lipid storage phenotype in NPC1 neurons and hepatocytes has been associated with impaired autophagy.^35^ We, therefore, investigated whether an autophagy defect in NPC1 macrophages affects bacterial handling capacity and compared with CD in carriers of NOD2 variants (figure 2A).

We developed a bacterial handling assay in MDM as macrophages are indispensable for granuloma formation and express NOD2. We initially investigated to what extent prototypic CD-associated NOD2 variants influence MDP-dependent bacterial killing in our gentamicin protection assay. Consistent with previous studies,^11^ ^36^ MDM from patients with IBD carrying homozygous or two heterozygous NOD2 variants showed a defect in MDP-dependent bacterial handling of *S. typhimurium* (figure 2B, left) as well as AIEC (figure 2B, right), a strain previously implicated in CD pathogenesis.^30^ ^37^ ^38^

Interestingly, and in contrast to previously described assays that assessed NOD2 activation in dendritic cells,^11^ this assay showed an MDP-specific bacterial killing effect in healthy control MDM that was not dependent on Toll-like receptor (TLR) or cytokine co-stimulation (figure 2C, D). Furthermore, an inactive isomer of MDP (ddMDP) was unable to reduce CFU and blocking of NOD1 signalling with ML-130 (Nodinitib-1) did not inhibit bacterial killing, indicating that this mechanism involves NOD2 (figure 2E and see online supplementary figure S3).

Despite impaired bacterial handling in all patients with variant NOD2, the ability to elicit an MDP-dependent cytokine response was only affected in cells from patients expressing homozygous 1007fs but no other homozygous or compound heterozygous NOD2 variants (figure 2F and online supplementary figure S4). Activation of a separate NF-κB pathway with LPS resulted in normal levels of TNF in all tested individuals. These results indicate that NOD2 variants impair bacterial degradation and have a differential effect on cytokine responses.

### XIAP-deficient granulomatous IBD is associated with defects in antibacterial autophagy

To further investigate whether defects in bacterial handling are commonly related to granulomatous intestinal inflammation, we studied individuals with loss-of-function mutations in XIAP. Patients with XIAP deficiency develop CD-like fistulising intestinal inflammation with granuloma formation (figure 3A).^17–19^ XIAP-associated IBD can be cured by stem cell transplantation,^18^ suggesting a substantial defect in the haematopoietic compartment. Although XIAP is an essential transducer of NOD2-dependent cytokine signalling,^24^ its role in bacterial handling has not been studied in humans. Furthermore, infection models in XIAP-deficient mice are inconclusive. Whereas they were not susceptible to *S. typhimurium* infection, they exhibited substantially decreased survival after *Listeria monocytogenes* infection.^22^ ^39^

**Figure 3:**
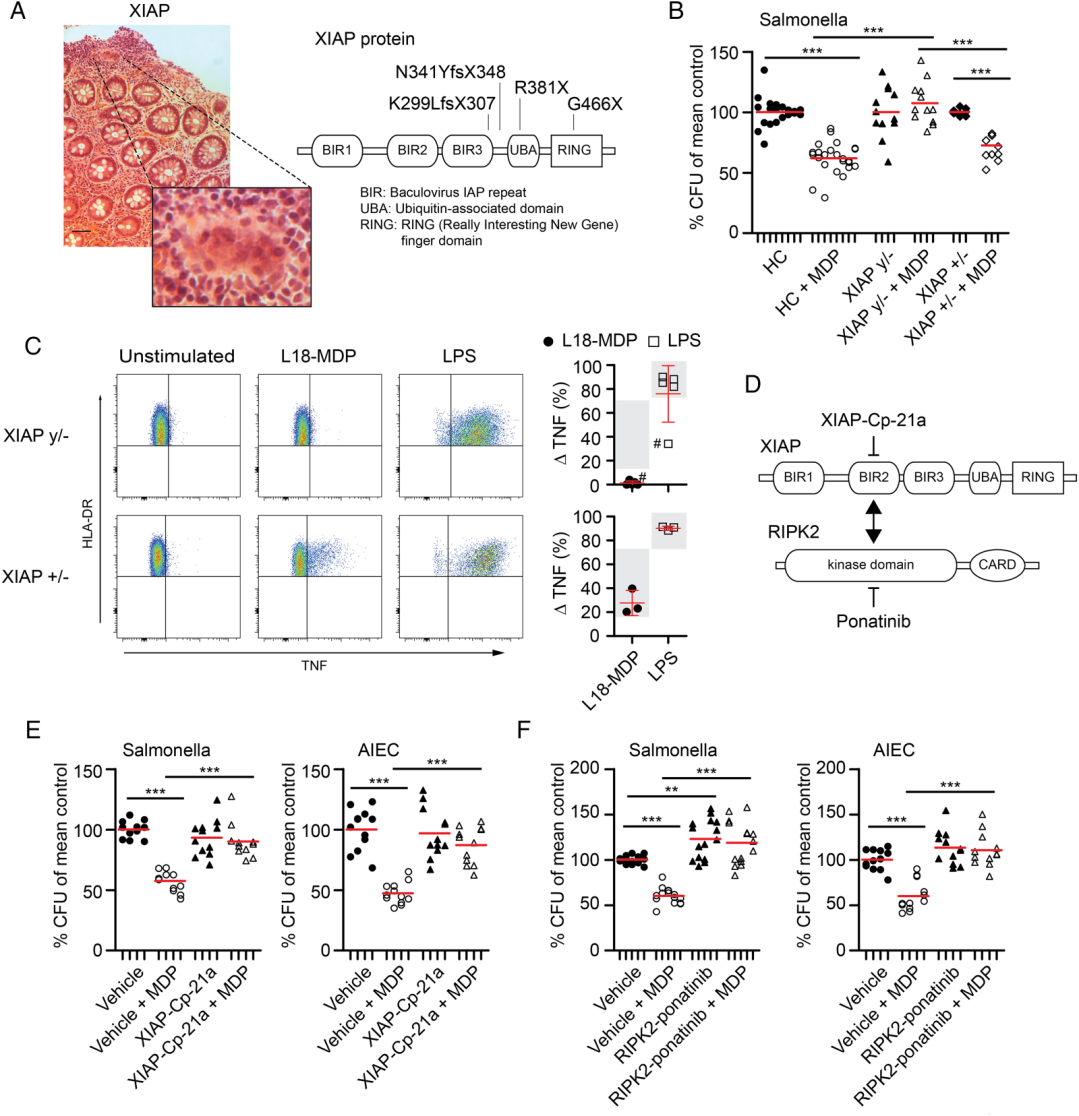
X-linked inhibitor of apoptosis (XIAP) and receptor-interacting kinase 2 (RIPK2) mediate activation of pro-inflammatory cytokines and killing of intracellular bacteria. (A) Granulomatous colitis in XIAP deficiency (H&E stain). Enlarged view shows granuloma at higher magnification. Scale bar, 50 μm. Schematic of XIAP protein indicating patient mutations. (B) Gentamicin protection assay with monocyte-derived macrophages (MDM) from healthy controls (n=6 HC), male XIAP-deficient patients (n=4 XIAP y/−) and female carriers (n=3 XIAP+/−) pre-stimulated with or without muramyl dipeptide (MDP). Individual results are shown as indicated by ticks on x-axes. For each donor, conditions were tested in three parallel infection experiments and colony-forming units (CFU) were normalised to individual CFU without MDP stimulation. Red bar represents mean. (C) Representative flow cytometry plots and quantification of NOD2 and TLR4 responses in HLA-DR^+^CD14^+^ monocytes (n=5 XIAP y/−, n=3 XIAP+/−). ^#^Bacteria-contaminated culture medium caused high baseline production of tumour necrosis factor (TNF) in indicated patient. Mean±SD, grey background indicates normal range. (D) Schematic of XIAP-RIPK2 interaction with small-molecule inhibitors. (E) Gentamicin protection assay performed as in (B) with healthy control MDM. Cells were cultured in the presence or absence of XIAP-Cp-21a (1 µM) and infected with *Salmonella typhimurium* (left, n=4) or adherent-invasive *Escherichia coli* (AIEC) (right, n=4). (F) Healthy control MDM were assayed as in (B) in the presence or absence of ponatinib and infected with *S. typhimurium* (left, n=5) or AIEC (right, n=4). (G) Induction of TNF in healthy donor monocytes pre-incubated with the XIAP-Cp-21a (1 μM) or ponatinib and activated with L18-MDP or LPS (100 ng/mL). (H) Purification of endogenous ubiquitin (Ub) conjugates using tandem ubiquitin binding entities (TUBE) in lysates of MDM pretreated with XIAP-Cp-21a. Purified material and lysate was examined by immunoblotting for the indicated proteins. p Values were throughout determined using Mann–Whitney U test. *p<0.05, **p<0.01, ***p<0.001.

**Figure 3:**
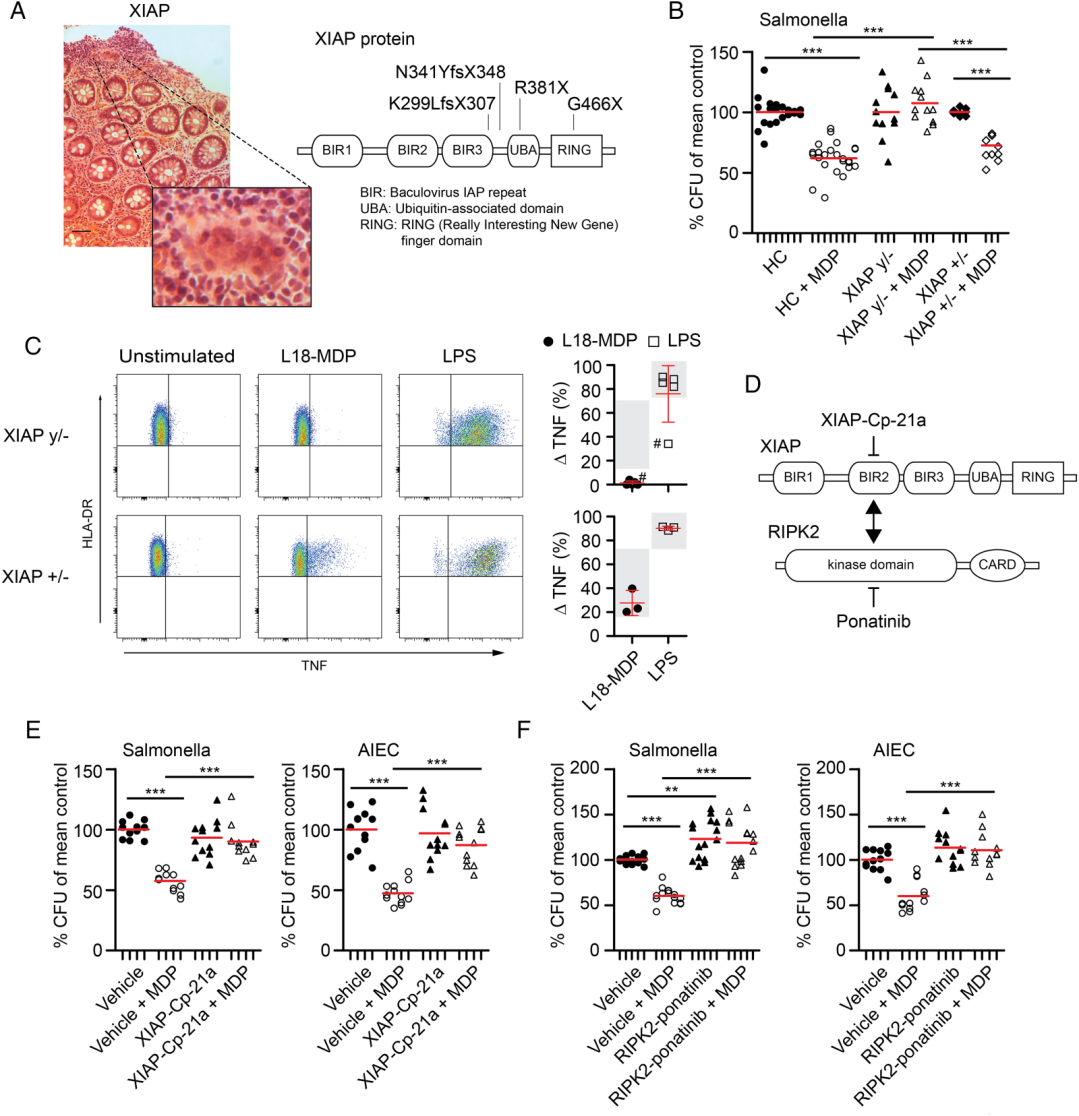
Continued

Due to the X-linked inheritance, we obtained MDM from XIAP-deficient males and female carriers of XIAP-defects. All mutations disrupted XIAP's C-terminal RING domain, which confers Ub-ligase activity (figure 3A).^23^ As shown in figure 3B,C, XIAP-deficient macrophages failed to kill bacteria in an MDP-dependent manner and showed disrupted TNF induction. In contrast, female carriers of a defective *XIAP* gene showed normal TNF production and normal bacterial handling (figure 3B, C). Under constitutive conditions, bacterial handling in XIAP-deficient macrophages did not differ from healthy controls (data not shown).

One mutational hotspot in XIAP-deficient patients is the BIR2 domain that mediates binding of XIAP to the adapter kinase RIPK2.^24^ To mimic the functional consequences of mutations in the XIAP-BIR2 domain, we used compound 21a (XIAP-Cp-21a), which selectively targets this domain (figure 3D).^28^ Accordingly, XIAP-Cp-21a impaired MDP-dependent bacterial killing and TNF production to a similar extent as observed in XIAP-deficient males (figure 3B, E). XIAP-Cp-21a directly interfered with initial pathway activation and impaired MDP-induced RIPK2 ubiquitination (figure 3H).

Since NOD2 and XIAP functionally converge on the adapter kinase RIPK2, we next investigated its role for bacterial killing in macrophages using the RIPK2 tyrosine kinase inhibitor ponatinib.^40^ ^41^ Consistent with a role of RIPK2 as part of the NOD2-XIAP complex, ponatinib completely abrogated MDP-induced TNF production and bacterial killing but showed no effect on LPS-dependent TNF upregulation (figure 3F, G).

### The lysosomal storage disease NPC1 impairs bacterial handling in macrophages

Since bacterial handling is defective in classical NOD2-CD and XIAP-associated CD, we evaluated whether lysosomal lipid storage affects bacterial handling in MDM of patients with NPC1 or MDM with pharmacologically induced NPC1 storage phenotype.

**Figure 4:**
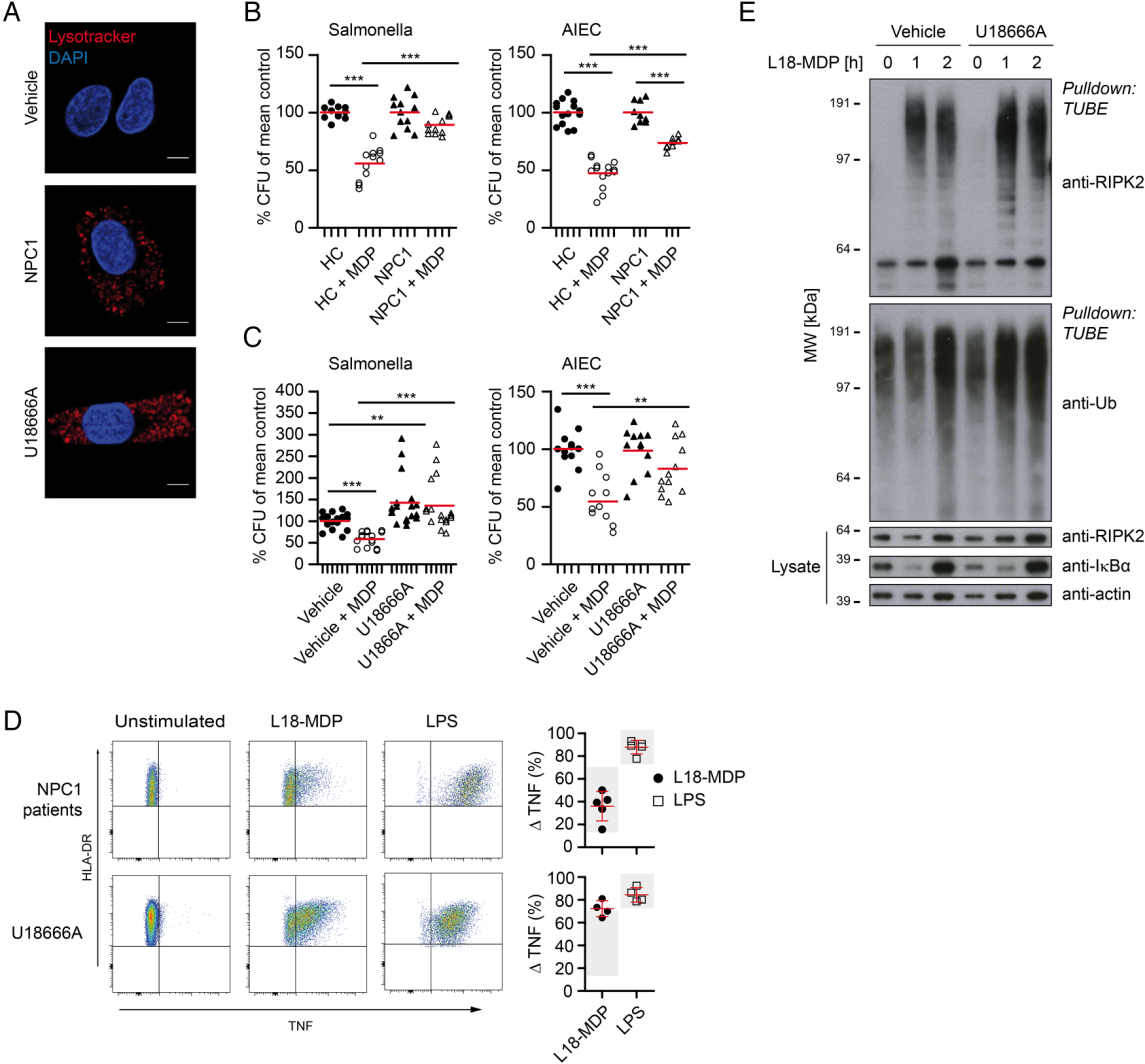
Niemann–Pick disease type C1 (NPC1) lysosomal lipid storage disease causes ineffective pathogen clearance despite functional cytokine pathways. (A) Lysosomal lipid storage phenotype in vehicle-treated, U18666A-treated and primary NPC1 monocyte-derived macrophages (MDM). Scale bar, 5 µm. (B) Bacterial killing assay with healthy (n=4–5) and NPC1 patient (n=3–4) MDM and infected with *Salmonella typhimurium* (left) or adherent-invasive *Escherichia coli* (AIEC) (right). Individual results are shown as indicated by ticks on x-axes. For each donor, conditions were tested in three parallel infection experiments and colony-forming units (CFU) were normalised to individual CFU without muramyl dipeptide (MDP) stimulation. Red bar represents mean. (C) Cells were assayed as in (B). MDM treated with or without U18666A and MDP pre-stimulation were exposed to *S. typhimurium* (left, n=4) or AIEC (right, n=4). (D) Representative flow cytometry plots and quantification of tumour necrosis factor (TNF) response in primary NPC1 (n=5) or U18666A-treated (n=4) HLA-DR^+^CD14^+^ monocytes following NOD2 or TLR4 stimulation. Mean±SD, grey background indicates normal range calculated from all measured healthy donors. (E) Purification of endogenous ubiquitin (Ub) conjugates using tandem ubiquitin binding entities (TUBE) in lysates of U18666A-treated and control MDM. Purified material and lysate was examined by immunoblotting for indicated proteins. p Values were throughout calculated using Mann–Whitney U test. *p<0.05, **p<0.01, ***p<0.001.

Primary NPC1 patient MDM showed enlarged lysosomes and the efficiency of MDP-dependent bacterial killing was diminished (figure 4A, B and online supplementary figure S5). This defect was evident for *S. typhimurium* (figure 4B, left) and, to a lesser extent, for AIEC (figure 4B, right). To verify our results, we modelled the NPC1 phenotype in vitro. Culture of MDM in the presence of U18666A for 48 h causes an accumulation of lipids in the late endosomal and lysosomal compartment^2^ and phenocopies the lipid storage disease (figure 4A). In our MDM bacterial handling assay, U18666A treatment increased baseline level of intracellular bacteria compared with untreated MDM and impaired MDP-mediated control of bacteria (figure 4C).

To investigate whether the impaired bacterial handling was due to defective NOD2 signal initiation, we tested the response to MDP by flow cytometry. Defects in NPC1 did neither abrogate NOD2 nor TLR signalling (figure 4D) nor was the stimulation affected by miglustat (see online supplementary figure S6). Indeed, stimulation of U18666A-treated cells with bacterial ligands led to an enhanced production of pro-inflammatory cytokines (figure 4D and online supplementary figure S7). Furthermore, we observed functional ubiquitination of RIPK2 in U18666A-treated MDM, indicating that the signalling complex formed by NOD2-RIPK2-XIAP is functionally intact (figure 4E).

Altogether, these data suggest that NOD2-RIPK2-XIAP form a signal initiation complex in macrophages, leading to RIPK2 ubiquitination and subsequent activation of downstream NF-κB signalling as indicated by TNF expression as well as innate immunity against intracellular pathogens. However, NPC1 mutations cause a defect in bacterial handling, including NOD2-mediated xenophagy without affecting NOD2-dependent TNF expression.

### Defective autophagy in NPC1 impairs clearance of intracellular bacteria

This prompted us to investigate the mechanism of bacterial handling defect in patients with NPC1. We first excluded differences in bacterial uptake of GFP-expressing *S. typhimurium* (GFP-*S. typhimurium*) in U18666A-MDM and untreated-MDM and found similar infection rates (figure 5A, left). However, in U18666A-MDM, the GFP signal increased with time, indicating normal phagocytosis but confirming increased bacterial survival (figure 5A, right). Similarly, there was no difference in uptake of non-viable fragmented *E. coli* particles within 1 h (see online supplementary figure S8). As *E. coli* and *S. typhimurium* have been shown to be sensitive to bacterial killing mediated by reactive oxygen species (ROS), we also excluded differences in the production of ROS after phorbol myristate acetate (PMA) stimulation (see online supplementary figure S9).

**Figure 5:**
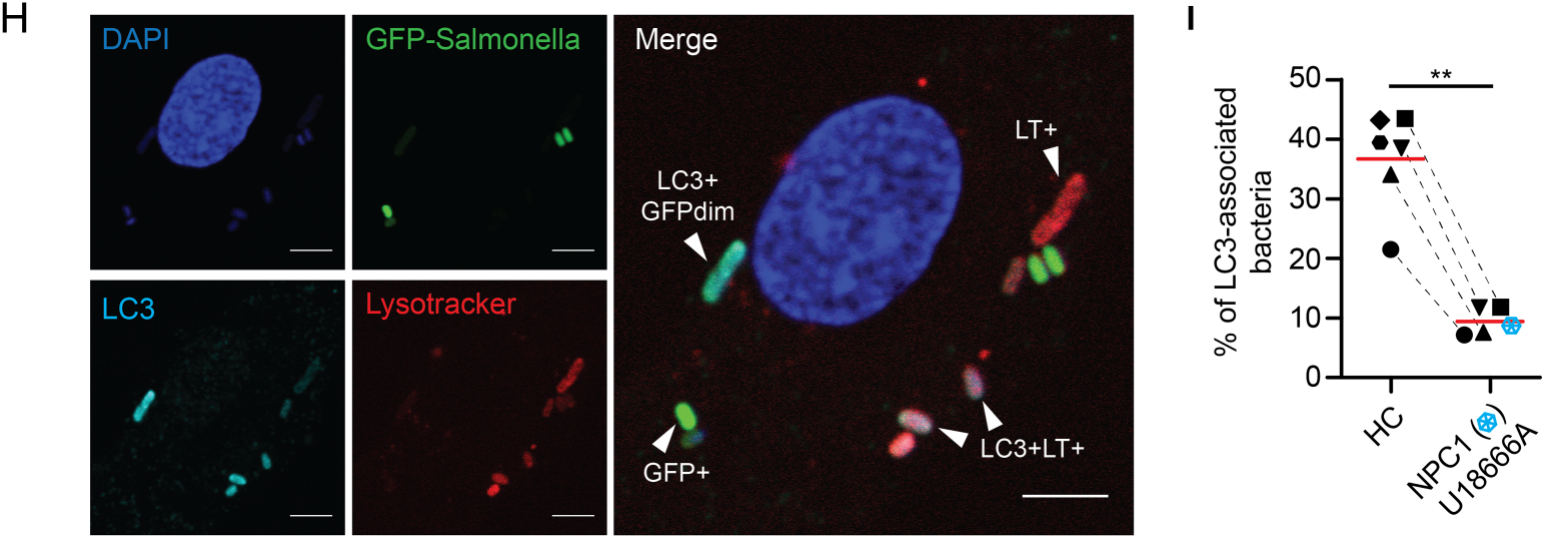
Increased bacterial colonisation of Niemann–Pick disease type C1 (NPC1) macrophages is caused by impaired antibacterial autophagy. (A) Flow cytometric analysis of green fluorescent protein (GFP) signal in healthy control monocyte-derived macrophages (MDM) treated with or without U18666A and challenged with GFP-*Salmonella typhimurium* for 1 h (infection, left) or incubated for one additional hour with gentamicin-containing medium (bacterial clearance, right). External bacteria were quenched with trypan blue. (B) Healthy control MDM were treated with or without U18666A followed by immunoblotting with anti-LC3, anti-p62 and anti-actin. Protein levels of LC3 and p62 were semi-quantified by densitometry relative to actin and expressed as per cent increase to vehicle-treated condition. (C–G) Primary NPC1 mutant and healthy control MDM were infected with GFP-*S. typhimurium* for 1 h, followed by 1 h culture in gentamicin-containing medium supplemented with Lysotracker (LT) and DAPI staining*.* Analysis is based on MDM from two patients with NPC1 and four healthy donors. (C) Representative images of infected macrophages. Scale bar, 5 μm. (D) Quantification of total number of bacteria per macrophage. (E) Microscopic assessment of GFP-*S. typhimurium* according to co-localisation with lysosomes. (F) Percentage of bacteria found in the different stages of autophagic degradation. (G) Absolute numbers of bacteria per MDM for indicated groups. (H) Infection experiment performed as in (C). Additionally, following fixation, MDM were stained with anti-LC3 and co-localisation with bacteria quantified by confocal microscopy. (I) Analysis of LC3-decorated bacteria in primary NPC1 mutant MDM (blue symbol) compared with healthy donor MDM (n=2) or MDM treated with or without U18666A (n=4). Each symbol represents one individual. p Values were determined by Mann–Whitney U test. *p<0.05, **p<0.01, ***p<0.001.

**Figure 5:**
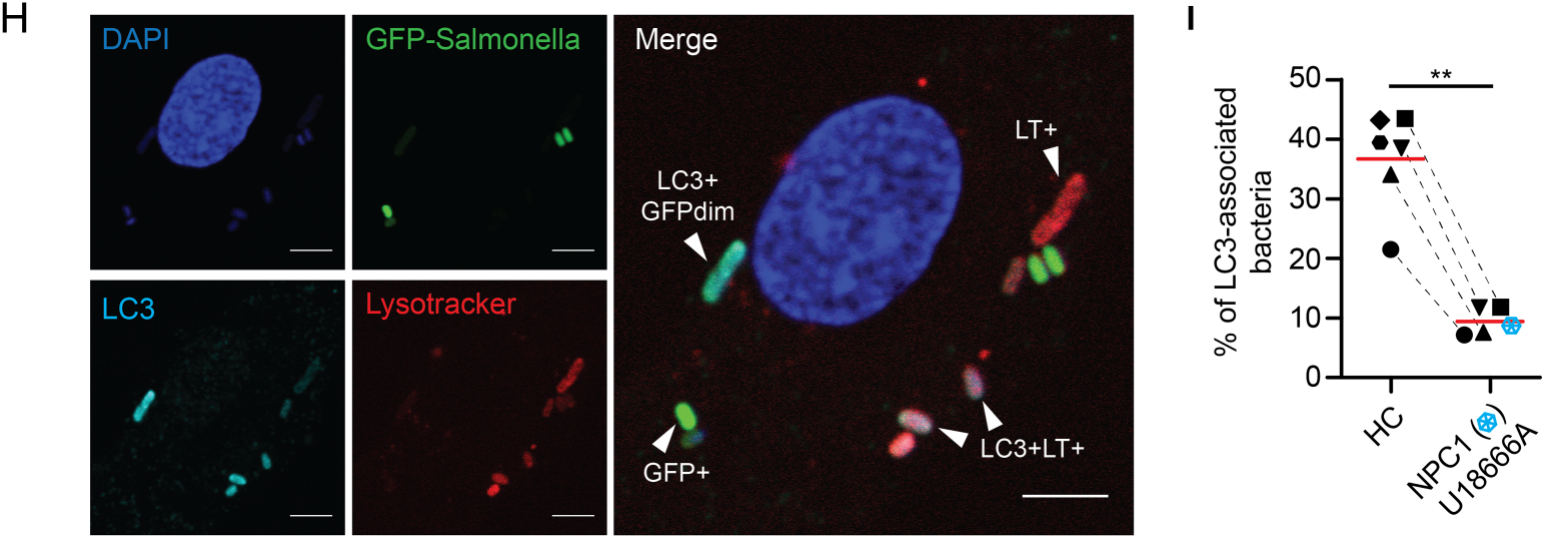
Continued

We next investigated whether the deregulation of autophagy affects NPC1 macrophage function. Of note, treatment with U18666A blocked steady-state autophagic flux in MDM, indicated by increased LC3 levels and decreased clearance of the autophagy substrate p62, which persisted even after exposure to *S. typhimurium* (figure 5B and online supplementary figure S10). This is consistent with findings in human and mouse NPC1 fibroblasts,^35^ showing increased LC3 punctuation suggestive of impaired maturation of autophagy-related vesicles (see online supplementary figure S11). This flux defect was not reversed by miglustat treatment (see online supplementary figure S11). Since lysosomal biogenesis and autophagy are also transcriptionally regulated, we investigated whether the major regulator transcription factor EB (TFEB) contributes to dysregulated autophagy. We found normal lysosomal biogenesis and nuclear translocation of TFEB in primary NPC1 fibroblasts and MDM with U18666A-induced storage phenotype, suggesting that this pathway does not play a major role (see online supplementary figure S12).

To gain further mechanistic insight, we infected MDM obtained from patients with NPC1and healthy controls with GFP-*S. typhimurium* and stained lysosomes with Lysotracker (LT) probe and cellular or bacterial DNA with 4′,6-diamidino-2-phenylindole (DAPI) (figure 5C). Consistent with previous results, NPC1 mutant MDM contained substantially more intracellular bacteria (figure 5D). Since bacteria lose their GFP signal in lysosomes, bacterial DAPI staining allowed continuous tracking of bacteria through different stages of degradation (figure 5E). We classified intracellular bacteria according to GFP and LT fluorescence and distinguished between four groups: (1) live GFP+ bacteria, (2) non-degraded GFP+ bacteria localising to lysosomes, (3) degraded GFP-bacteria localising to lysosomes and (4) DAPI+ bacterial remnants. In macrophages obtained from NPC1 patients, we observed significantly more live GFP+ bacteria that do not localise to lysosomes (figure 5F, G).

We initially assumed the reduced bacterial killing could be due to defective autophagosome-lysosome fusion and expected an increased number of LC3-decorated bacteria in the presence of defective lipid loaded lysosomes that do not fuse. Unexpectedly, in U18666A-MDM or primary NPC1 mutant MDM significantly fewer bacteria were LC3-labelled (figure 5H, I), whereas in WT-macrophages, a substantial number of intracellular bacteria can be found in LC3-associated vesicles indicative of antibacterial autophagy (figure 5H, I). This suggested that the defect in NPC1 impairs a critical stage of phagosome-autophagosome transition between bacterial sensing and bacterial degradation. Indeed, proteomic raw data of isolated phagosomes suggest that NPC1 is already expressed in early phagosomes of human and murine phagocytes.^42–44^

### Induction of autophagy rescues bacterial handling in NPC1

**Figure 6:**
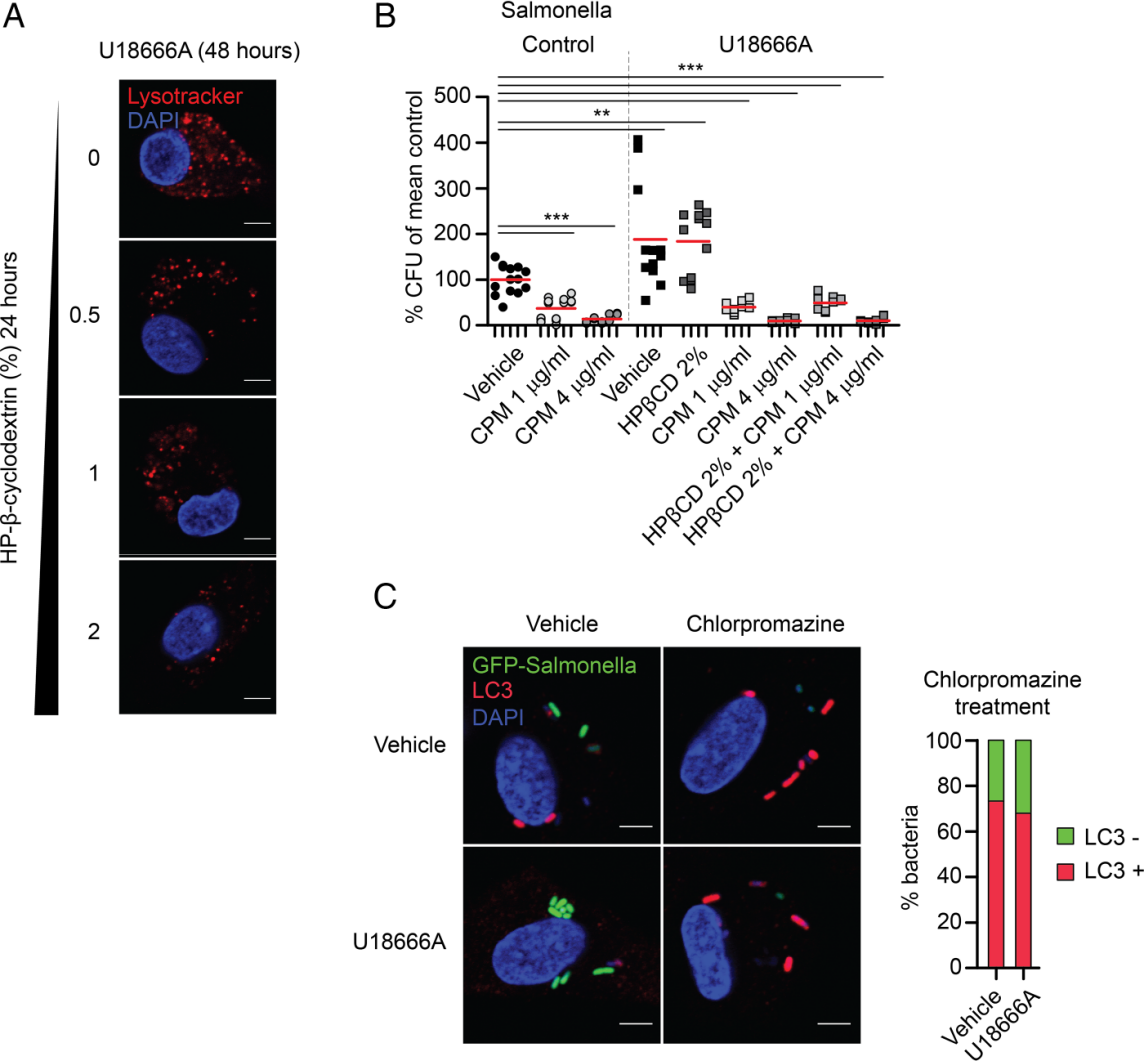
Induction of autophagy with chlorpromazine overcomes bacterial killing defect in Niemann–Pick disease type C1 (NPC1). (A) U18666A-treated monocyte-derived macrophages (MDM) were cultured in 0.5–2% of HP-β-cyclodextrin (HPβCD) for last 24 h of U18666A treatment (48 h) and stained with Lysotracker red. Scale bar, 5 μm. (B) Gentamicin protection assay in vehicle- or U18666A-MDM pre-treated with chlorpromazine for 3 h before infection with *Salmonella typhimurium*, n=4. Treatment of U18666A-MDM with 2% HPβCD was performed for last 24 h in the presence of U18666A (48 h). Individual results are shown as indicated by ticks on x-axes. For each donor, experimental conditions were tested in three parallel infection experiments and colony-forming units (CFU) were normalised to individual vehicle-treated control. Red bar represents mean. p Values were determined by Mann–Whitney U test comparing results to vehicle-treated control, ***p<0.001. (C) Immunofluorescence staining with anti-LC3 of untreated or U18666A-treated MDM pre-incubated with chlorpromazine for 3 h before exposure to green fluorescent protein (GFP)-*S. typhimurium.* Scale bar, 5 μm. Quantification of LC3-decorated bacteria in chlorpromazine conditions was performed in one representative experiment.

Potential treatment strategies for the neurodegenerative disease NPC1 focused until recently on the depletion of cholesterol from the lysosomal/late-endosomal compartment, for example, by means of HP-β-cyclodextrin (HPβCD).^45^ HPβCD has been used successfully to treat neuron lysosomal storage in vitro and in vivo. Since HPβCD does not pass the blood–brain barrier, we reasoned that it might be a peripherally acting agent beneficial for restoring macrophage function. As expected, HPβCD reduced the lysosomal volume of U18666A-MDM dose-dependently (figure 6A). However, despite effective cholesterol depletion and normalisation of the lysosomal volume in cells, this treatment did not rescue the dysfunctional bacterial handling in U18666A-MDM (figure 6B). Most recently, autophagy induction was suggested as a therapeutic strategy for IBD and neurodegenerative diseases.^46^ ^47^ We observed that treatment of U18666A-MDM with the autophagy enhancer chlorpromazine restored the defect in autophagic flux and resulted in normal bacterial degradation (figure 6B). Treatment of human MDM with chlorpromazine led to dose-dependent increase in LC3-mediated bacterial clearance (figure 6B, C). The differential effects of HPβCD versus chlorpromazine in macrophages argue again for a critical role of NPC1 in autophagy, affecting autophagosome function upstream of the lysosomal storage defect. Other inducers of autophagy, like rapamycin^11^ or trehalose,^48^ did either not improve bacterial handling in U18666A-MDM or were associated with substantial pro-inflammatory cytokine induction (see online supplementary figure S13).

## Discussion

**Figure 7:**
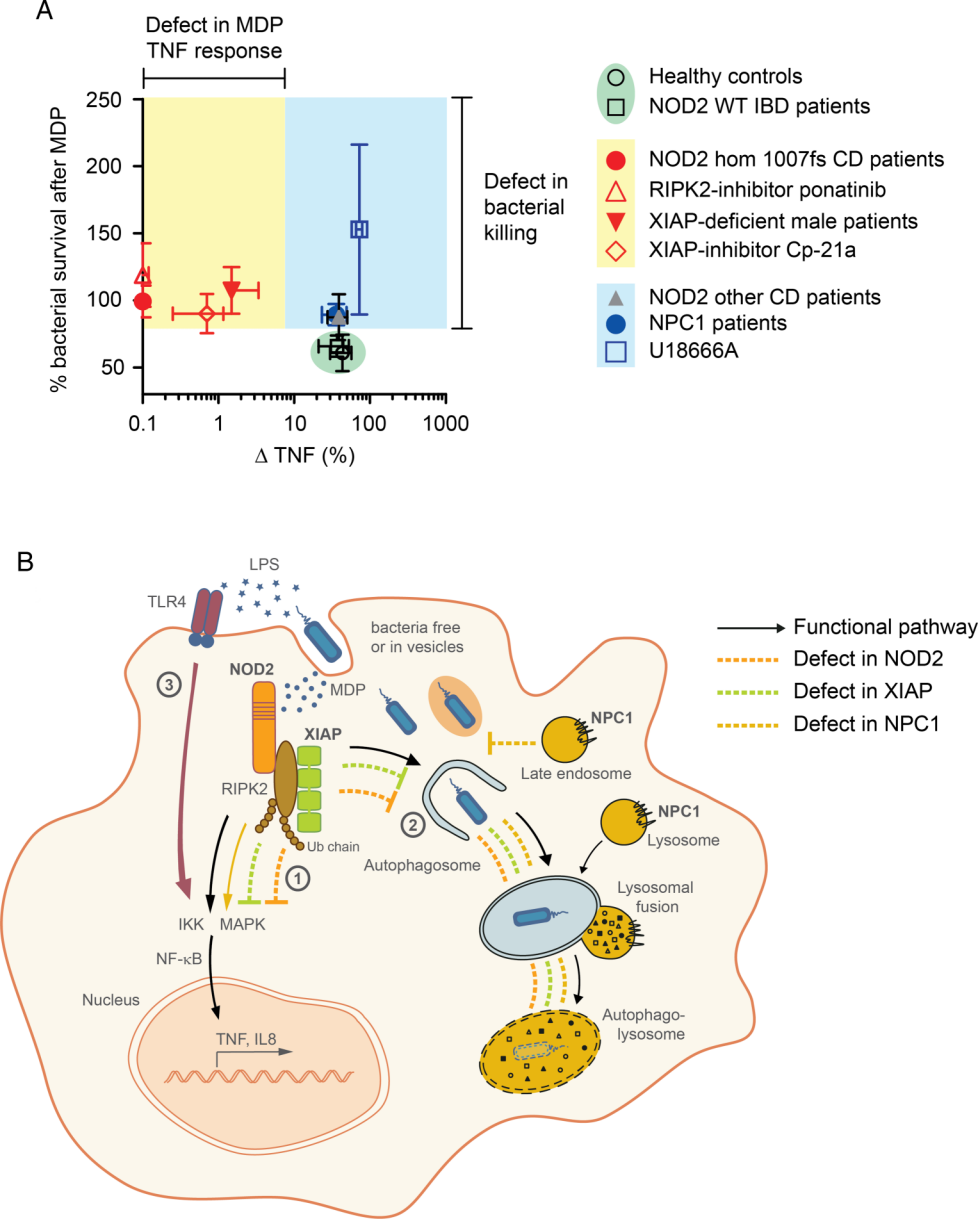
Summary figure and graphical abstract. (A) Summary figure of tumour necrosis factor (TNF) response and bacterial killing following activation of nucleotide-binding oligomerization domain-containing protein 2 (NOD2) pathway. Data represent mean±SD of results obtained from patients or inhibitors. (B) Graphical abstract summarising the functional mechanisms. NOD2-RIPK2-XIAP complex activates NF-κB-dependent signalling pathways (1) and induces clearance of cytosolic bacteria that requires functional autophagic-lysosomal interplay (2). Failure in prompt removal of invading bacteria may result in accumulation of bacteria leading to chronic stimulation of pattern-recognition receptors (eg, TLR4) by bacterial products causing a net NF-κB activation and consequently chronic inflammation (3). WT, wild-type.

We identify a group of patients with NPC1 mutations who develop severe early-onset CD-like intestinal inflammation with granuloma formation and perianal disease that cannot be distinguished from CD. We provide a mechanism of impaired bacterial handling in NPC1 that conceptually links NPC1-IBD to other genetic defects associated with granulomatous intestinal inflammation, NOD2 and XIAP. We confirm that XIAP deficiency, a cause of monogenic IBD with granuloma, does disrupt NOD2-mediated cytokine production^17–19^ and provide novel evidence that it disturbs NOD2-dependent xenophagy. Whereas mutations in NOD2 and XIAP impair the initiation of autophagic elimination of intracellular bacteria, NPC1 impairs autophagosome function (figure 7A). Although these molecules act at different checkpoints along the antibacterial autophagy or cytokine pathway (figure 7B), genetic variation in NOD2, XIAP and NPC1 results in a similar intestinal phenotype. Thus, heterogeneous genetic defects highlight bacterial handling as a likely defect of general importance in CD immune-pathogenesis with multiple cassettes or pathways contributing.

There is good genetic evidence that susceptibility to polygenic CD is associated with several common variants affecting autophagy.^49^ Variants in NOD2 functionally link bacterial recognition to selective antimicrobial autophagy involving ATG16L1.^11^ ^13^ ^36^ ^50^ Whereas previous studies might have predicted the effect of XIAP deficiency on NOD2-dependent xenophagy, the link to NPC1 represents a novel IBD susceptibility gene. Although spontaneous colitis has not been described in animal models of NPC, several reasons suggest causality in human NPC1-IBD rather than mere coincidence: (1) the association of NPC1 with CD-like immunopathology but not UC, (2) the early paediatric onset of symptoms with severe phenotype and (3) the reproducible finding among many centres making a reporting bias very unlikely.

Based on large cohort of patients with NPC in Manchester with approximately 150 patients, we were able to estimate a 3–7% penetrance of IBD in patients with NPC. Although this seems low, it should be seen in perspective that the penetrance of IBD in patients with one of the three most common NOD2 mutations (homozygous and compound heterozygous) is only about 1.5%.^51^ Indeed, as patients with NPC1 have a shorter life expectancy,^1^ the true impact of NPC1 deficiency for the development of IBD may have even been underestimated.

Based on our studies of three different human genotypes with CD-like immunopathology, we dissected MDP-dependent cytokine signalling and bacterial killing, which are parallel and partially independent pathways downstream of the NOD2-RIPK2-XIAP initiation complex. Indeed, loss of function in NPC1 impairs the control of bacterial handling without loss of NF-κB-driven cytokine release. Our data imply that defects in NPC1 impair the process of steady-state autophagosome maturation and affect autophagic trafficking of bacteria to lysosomes in macrophages. Since bacteria do not accumulate in LC3+ autophagosomes, we speculate that NPC1 might be involved upstream in phagosome-autophagosome transition. This mechanism becomes more obvious in the defence against *S. typhimurium* compared with AIEC since *S. typhimurium* is a facultative intracellular pathogen and clearance from the host strongly depends on autophagy.^52^ ^53^ The described immune dysfunction extents the role of NPC1 for autophagy beyond hepatic or neuronal cell defects with lipid storage disease.^35^ ^54^

Our results suggest that excessive inflammation seen in IBD is not only a result of dysfunctional NF-κB signalling in macrophages but secondary due to insufficient removal of intruding bacteria. As a functional consequence, these pathogens serve as a constant stimulus on other pattern recognition receptors with intact signalling cascades, for example, toll-like receptors that finally lead to increased cytokine production. Current treatment strategies in CD aim to suppress this active inflammation and use immunosuppressive strategies to maintain remission.^55^ Our data provide a strong rationale to develop drugs that target the defective bacterial defence and enhance macrophage clearance function. We used chlorpromazine since macrophages are relative insensitive to rapamycin^56^ and our preliminary data suggest that these drugs have a different mechanism of action. Chlorpromazine represents a group of autophagy inducers that includes several US Food and Drug Administration-approved drugs^57^ and has previously been identified to improve cellular defence against intracellular bacteria in mouse bone marrow-derived macrophages.^58^ The mechanism of action for chlorpromazine and the related group of autophagy-inducing substances with a joint N^10^-substituted phenoxazine scaffold needs to be clarified. It will be interesting to evaluate whether autophagy-inducing therapies have effects during active inflammation or rather in maintaining remission.

In conclusion, our data imply that impaired bacterial handling underlies several genetic defects associated with granulomatous colitis and therapeutic modification of bacterial handling is a potential treatment option in IBD.
