# Supplementary material for: Impaired antibacterial autophagy links granulomatous intestinal inflammation in Niemann–Pick disease type C1 and XIAP deficiency with NOD2 variants in Crohn's disease
Source: Gut. 2016 Mar 7;66(6):1060–73. doi: 10.1136/gutjnl-2015-310382 (PMC5532464; doi:10.1136/gutjnl-2015-310382)
Supplement: Supplementary data [file gutjnl-2015-310382supp001.pdf]

Supplementary Materials for:

Impaired anti-bacterial autophagy links granulomatous intestinal inflammation in Niemann-Pick disease type C1 and XIAP deficiency with NOD2 variants in Crohn's disease

Tobias Schwerd[1], Sumeet Pandey[1], Huei-Ting Yang[1], Katrin Bagola[2], Elisabeth Jameson[3], Jonathan Jung[1], Robin H. Lachmann[4], Neil Shah[5], Smita Y. Patel[6], Claire Booth[7], Heiko Runz[8], Gesche Düker[9], Ruth Bettels[10], Marianne Rohrbach[11], Subra Kugathasan[12], Helen Chapel[6], Satish Keshav[1], Abdul Elkadri[13,14], Nick Platt[15], Alexio M. Muise[13,14], Sibylle Koletzko[16], Ramnik J. Xavier[17], Thorsten Marquardt[10], Fiona Powrie[1], James E. Wraith[3], Mads Gyrd-Hansen[2], Frances M. Platt[15], Holm H. Uhlig[1,18]*

*Correspondence author. Email: holm.uhlig@ndm.ox.ac.uk (H.H.U.)

The PDF file includes:

Supplementary Methods

Supplementary Figures 1-13

Supplementary Tables 1-3

Supplementary References

Supplementary Methods

Research subjects

NPC1 patients

Patient phenotype data were captured using a structured survey including demographics, family history, diagnosis of NPC, NPC genotype and IBD-like immunopathology, disease location according to Paris classification [1], extra-intestinal manifestations, infections, operations, miglustat therapy and treatment of IBD. Data from clinical centres, ehealthMe database and the Center of Disease Control (CDC) were compared to avoid multiple reporting.

Cell lines

Human fibroblasts and murine macrophage cell line RAW264.7 stably expressing TFEB-3xFLAG were cultured in DMEM (Sigma-Aldrich) supplemented with 10% FCS (Sigma-Aldrich). Healthy control human fibroblasts (GM05659) and NPC1 patient fibroblasts (GM17911) were obtained from Coriell Cell Repositories. TFEB-3xFLAG RAW264.7 cell line was a kind gift from M. Ferron and G. Karsenty and has been described previously [2].

Histology and H&E stain

Routine biopsies of patients were evaluated for histopathological features of IBD. Hematoxylin and eosin (H&E) or Periodic acid–Schiff (PAS) stains were carried out using routine methods.

Intracellular flow cytometry

Measurement and quantification of intracellular TNF in monocytes was performed as described in Ammann et al. [3] with minor modification: PBMC were cultured in RPMI supplemented with 10% FCS and a live-dead stain (Fixable Viability Dye, eBioscience) was included in the staining procedure. Antibodies against human TNF (MAb11, eBioscience), CD14 (M5E2) and HLA-DR (L243, both BioLegend) were used. Samples were acquired on the LSRFortessa flow cytometry system and data were analysed with FlowJo software (Version 10.0.6, Treestar). TNF expression was

analysed in single, live, HLA-DR^{+}, CD14^{+} monocytes. Induction of TNF (Δ TNF) was calculated as difference between the frequency of TNF-positive monocytes in unstimulated and stimulated conditions. Data represent mean±sd. Grey background indicates normal range (mean±2sd) calculated from all measured healthy individuals (n=25).

LC3 measurement by flow cytometry

LC3-levels in fibroblasts were assayed by flow cytometry with the Autophagy Detection Reagent Pack according to manufacturer's instructions (Merck Millipore).

Confocal microscopy

MDM were seeded at a density of 1 x 10^{5} cells in 8-well chamber slides (Sarstedt). The following morning cells were infected with freshly grown GFP-*S. typhimurium* at a MOI of 20 for one hour. Subsequently, cells were washed and treated for an additional hour with complete medium supplemented with 100 µg/ml gentamicin and for labelling of acidic cell compartments with 100 nM of the acidotropic dye Lysotracker Red or Deep Red (Invitrogen). After fixation with 2% paraformaldehyde, cellular and bacterial DNA was stained with DAPI and slides mounted with Vectashield mounting medium (Vector Laboratories). For staining of autophagy marker LC3, fixed cells were permeabilized with 0.1% Triton X-100 for 10 minutes and immunostained with anti-LC3 (clone PM036, MBL) and appropriate secondary antibodies (Alexa Fluor 568 goat anti-rabbit IgG, Life technologies). Images of infected MDM were acquired as z-stacks of multiple sections collected at 0.5 µm intervals at 63x magnification with a Zeiss 510 or 780 inverted confocal microscope (ZEN2009 or ZEN2011 software) and quantified with ImageJ software [4]. A minimum of 100 infected cells were evaluated for each experimental condition without image editing. For illustration of lysosome staining in MDM images were processed by Adobe Photoshop CS4.

For microscopic evaluation of TFEB nuclear translocation, TFEB-3xFLAG RAW264.7 cells were stimulated with Torin 1 (10 µM, 3 hours) or infected with *S. typhimurium* (MOI 20, 2 hours). Following fixation and permeabilization with 0.1% Triton X-100, cells were stained with mouse

monoclonal anti-FLAG (clone M2, Sigma-Aldrich) and secondary Alexa Fluor 568 goat anti-mouse IgG (Life technologies). Cell nuclei were counterstained with DAPI. Images of RAW264.7 macrophages were acquired at 63x magnification with a Zeiss 510 inverted confocal microscope (ZEN2009 software) and quantified with ImageJ software [4] without image editing. For illustration of TFEB nuclear translocation images were processed by Adobe Photoshop CS4.

Measurement of reactive oxygen species

Production of reactive oxygen species by MDM was assayed with the chemiluminescence probe L-012 (100 µM, Wako laboratories, Japan) in opaque white 96-well plates. Cells were activated with PMA (1 µg/ml, Sigma-Aldrich) in the presence or absence of diphenylene iodonium (DPI, 1 µM, Sigma-Aldrich), a general inhibitor of NADPH oxidases. Photo emission was recorded every 10 minutes with a plate reader (FLUOstar OPTIMA, BMG labtech).

Phagocytosis assay

Uptake of *E. coli* particles was studied with pHrodo Red *E. coli* bioparticles (Invitrogen) according to manufacturer's instructions. MDM, seeded in opaque black 96-well plates, were incubated for 30 minutes with bioparticles, washed with PBS and fresh medium supplemented with or without bafilomycin A1 (50 nM) was added. Fluorescence was determined 30 minutes later with a plate reader (FLUOstar OPTIMA, BMG labtech). Experiments were performed in triplicates.

Purification of endogenous Ubiquitin conjugates

Purification of endogenous Ubiquitin-conjugates was performed with Tandem Ubiquitin Binding Entities (TUBE) as described previously by Damgaard et al. [5]. In brief, MDM (5 x 10^6 cells) were lysed in 200 µl TUBE lysis buffer (20 mM Na2HPO4, 20 mM NaH2PO4, 2 mM EDTA, 1% Nonidet-P40, 1 mM DTT, 5 mM NEM, protease inhibitor and phosphatase inhibitor [Roche]) supplemented with 50 µg/ml purified GST-TUBE and incubated on ice for 30 minutes. After centrifugation for 15 minutes at 16,000g and 4°C, 5% of the supernatants were taken as input.

Remaining supernatant was incubated overnight at 4°C with glutathione sepharose 4Fast Flow (GE healthcare) and sepharose 6B (each 6 µl bed volume) mixed in 100 µl lysis buffer. Subsequent to washing of the sepharose with PBS-Tween (0.01%) and elution of bound material in Laemmli buffer, samples were analysed by SDS-PAGE and immunoblotting.

Cell fractionation

Cytoplasmic and nuclear protein fractions were separated according to manufacturer's instructions with NE-PER nuclear and cytoplasmic extraction reagents (Thermo Scientific) containing protease inhibitors (Roche). Nuclear and cytoplasmic proteins were separated by SDS-PAGE and analysed by immunoblotting.

Immunoblotting

Unless stated otherwise, cells were lysed in pH 7.5 buffer containing 50 mM Tris, 150 mM NaCl, 2 mM EDTA, 50 mM NaF, 1% Nonidet-P40 and 2 mM Na4P2O7 plus protease inhibitors (Roche). For SDS-PAGE, samples were separated on NuPAGE Bis-Tris gels with MOPS or MES running buffer (Life technologies) followed by immunoblotting according to standard procedures. The following antibodies were used: mouse monoclonal anti-RIPK2 (clone A10, sc-166765, Santa Cruz), mouse monoclonal anti-ubiquitin (clone Ubi-1, IMG-5021, Imgenex and clone FK2, D058-3, MBL), rabbit polyclonal anti-IκBα (#9242, Cell Signaling), rabbit monoclonal anti-LC3 (clone D11, #3868, Cell Signaling), rabbit monoclonal anti-p62 (clone D5E2, #8025, Cell Signaling), mouse monoclonal anti-p62 (clone 3/P62, #610833, BD Biosciences), rabbit polyclonal anti-TFEB (#4240, Cell Signaling), rabbit monoclonal anti-phospho-S6 Ribosomal Protein (Ser235/236) (clone D57.2.2E, #4858, Cell Signaling), rabbit monoclonal anti-Histone H3 (clone D1H2, #4499, Cell Signaling), mouse monoclonal anti-LAMP1 (clone H4A3, sc-20011, Santa Cruz), rabbit monoclonal anti-β-Tubulin (clone 9F3, #2128, Cell Signaling), mouse monoclonal anti-actin (clone C4, MAB1501, Millipore) and HRP-conjugated secondary antibodies (Cell Signaling, GE Healthcare).

Expression analysis and ELISA

Quantitative PCR was performed according to established standard protocols. Briefly, RNA was extracted using RNAeasy kit (Qiagen), transcribed in cDNA with High Capacity cDNA Reverse Transcription Kit (Applied Biosystems) and expression data were obtained using TaqMan primers (Life Technologies) for LAMP1 and LAMP2. Results were normalized to RPLP0 expression. Relative gene expression was calculated using the ΔCt method.

Secretion of TNF and IL-8 into the cell culture supernatant was determined by ELISA following manufacturer's protocol (Ready-Set-Go kits, ebioscience).

Supplementary Results

Supplementary figure 1

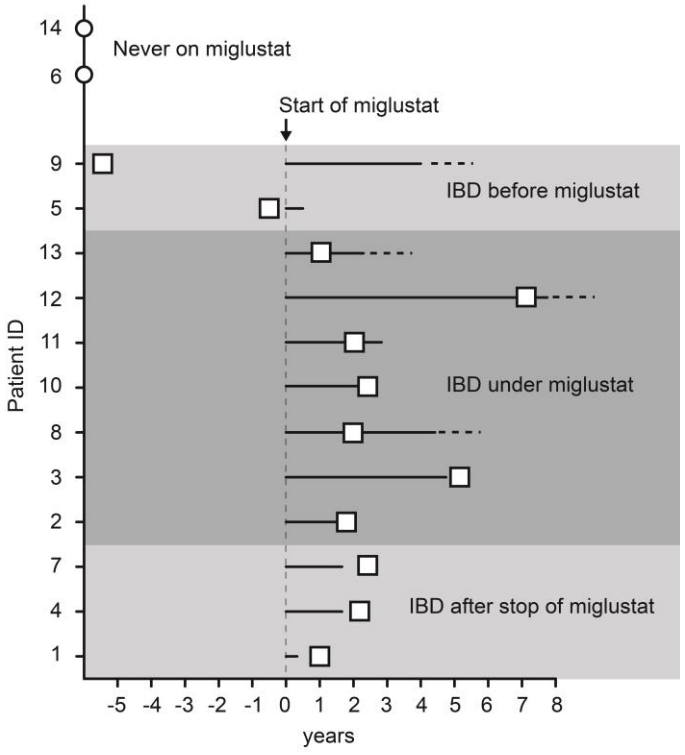

Supplementary figure 1 (related to figure 1). Diagnosis of IBD-like immunopathology in relation to miglustat treatment. Circles represent NPC1 patients with IBD who had never received miglustat and squares show onset of IBD in relation to treatment with miglustat. Arrow at time point "0" indicates start of miglustat. Horizontal dashed lines indicate continued use of miglustat after IBD diagnosis.

Supplementary figure 2

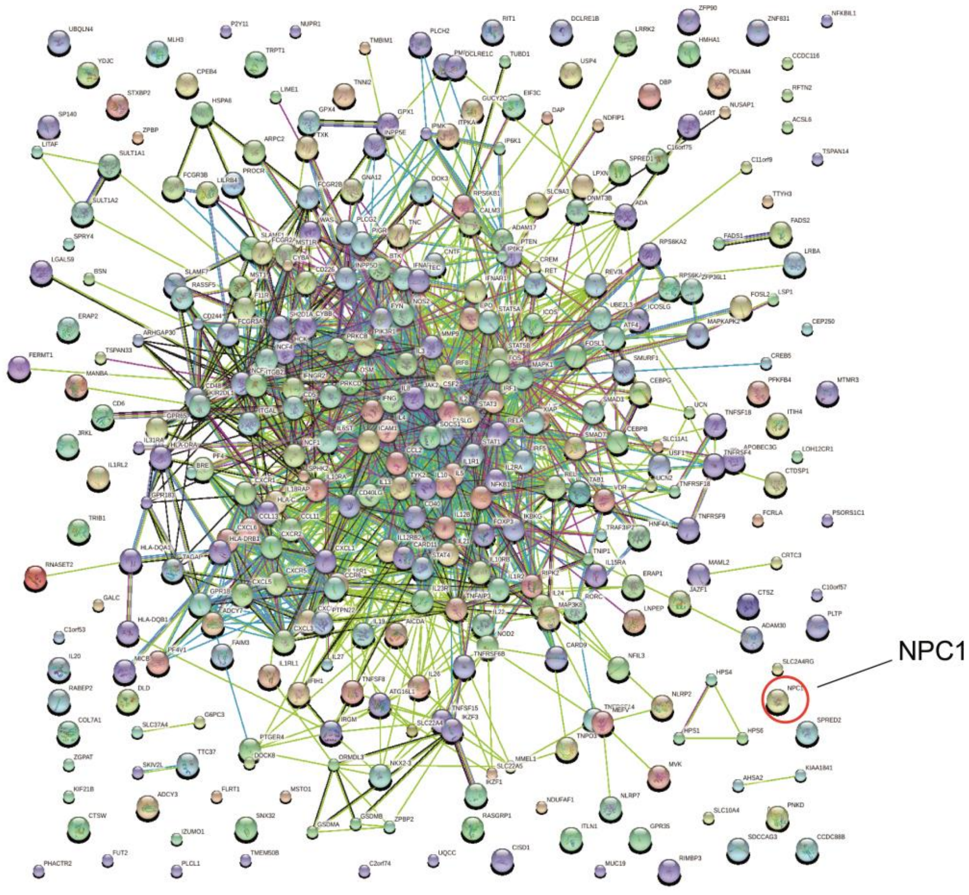

Supplementary figure 2 (related to figure 1). Interaction network of IBD susceptibility genes and NPC1 gene. Interaction network of 301 candidate genes within IBD susceptibility loci, 40 monogenic disorders associated with IBD-like immunopathology and *NPC1* gene. There is no evidence for direct interactions between NPC1 and other genes. Analysis based on String database (http://string-db.org).

Supplementary figure 3

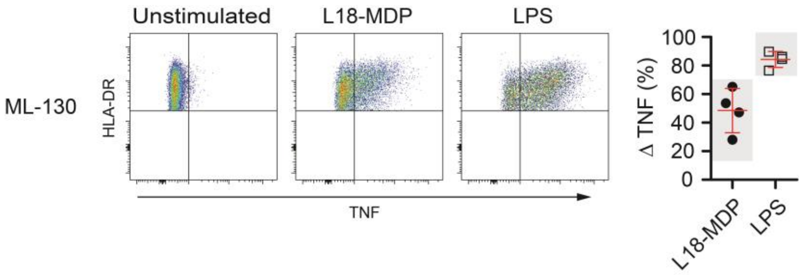

Supplementary figure 3 (related to figure 2). NOD1 inhibition and TNF production in monocytes following NOD2 or TLR4 stimulation. Representative flow cytometry plots and quantification of TNF up-regulation in healthy control monocytes (n=4) by L18-MDP and LPS in the presence of the small molecule NOD1 inhibitor ML 130. The induction of TNF by L18-MDP was comparable to stimulated monocytes of healthy controls (n=25), p=0.53, Mann-Whitney U test. Stimulation with LPS severed as positive control. Grey background indicates normal range (mean±2sd) calculated from all measured healthy individuals.

Supplementary figure 4

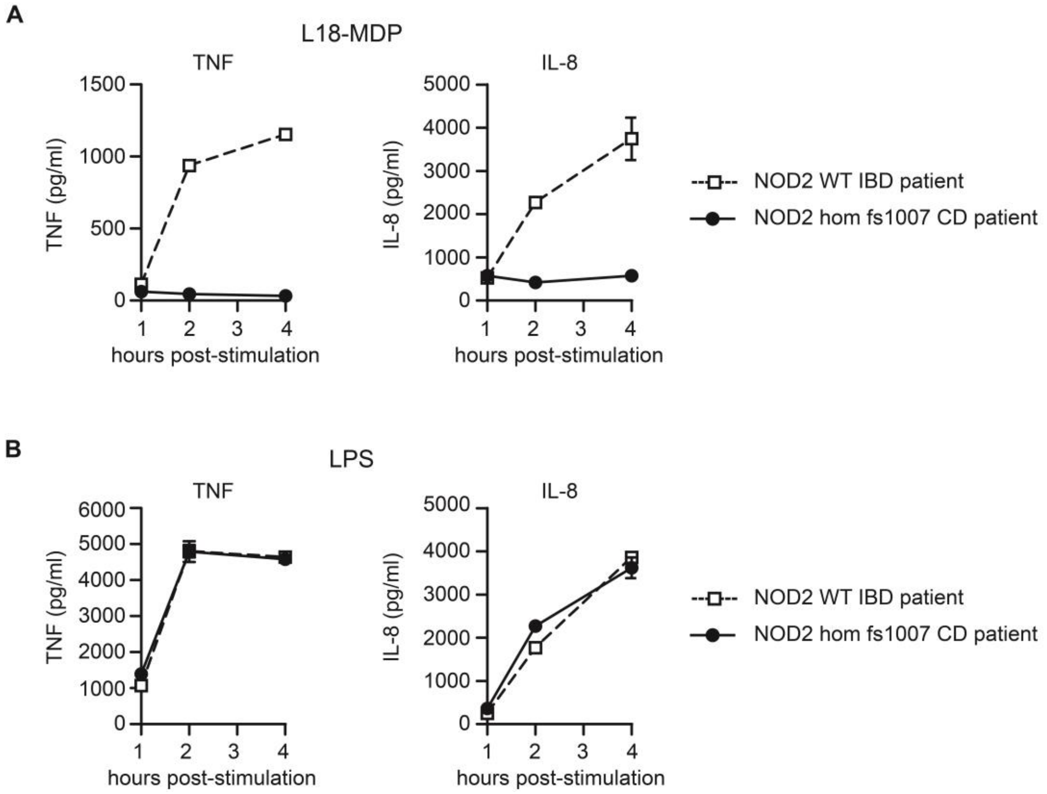

Supplementary figure 4 (related to figure 2). Abrogated cytokine secretion in macrophages expressing homozygous 1007fs NOD2. MDM generated from one CD patient with homozygous 1007fs NOD2 mutation (NOD2 hom fs1007) and one IBD patient with wild-type NOD2 (NOD2 WT) were stimulated with 200 ng/ml L18-MDP (**A**) or 100 ng/ml LPS (**B**) for the indicated time points. Supernatant was analysed for TNF and IL-8 by ELISA.

Supplementary figure 5

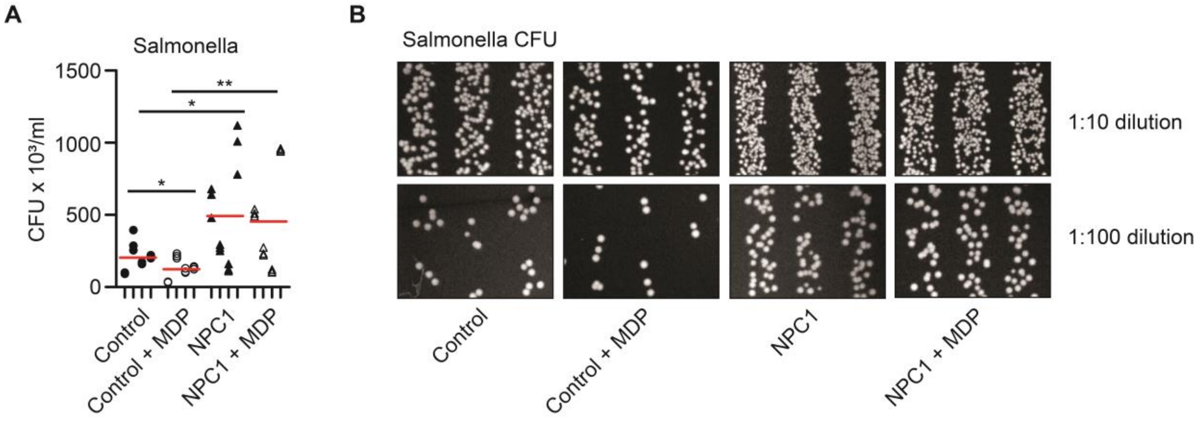

Supplementary figure 5 (related to figure 4). Absolute numbers of colony forming units in infected macrophages. NPC1 mutant macrophages contain more intracellular bacteria compared to control cells. (**A**) Absolute numbers of colony forming units (CFU) found in MDM challenged with *S. typhimurium.* For best comparability, bacterial colonies of NPC1 MDM are shown together with control MDM, plated and grown on the same square agar plate. Statistical significance was determined by Mann Whitney U test (*P<0.05, **P<0.01). After multiple testing with ANOVA and post-test correction with Bonferroni significant differences were found between control MDM and NPC1 MDM and between their MDP-treated conditions. (**B**) Representative pictures of LB agar plates showing *S. typhimurium* growing in triplicates. Intracellular bacteria are derived from lysates of MDM and are plated on square agar plates by track plate method.

Supplementary figure 6

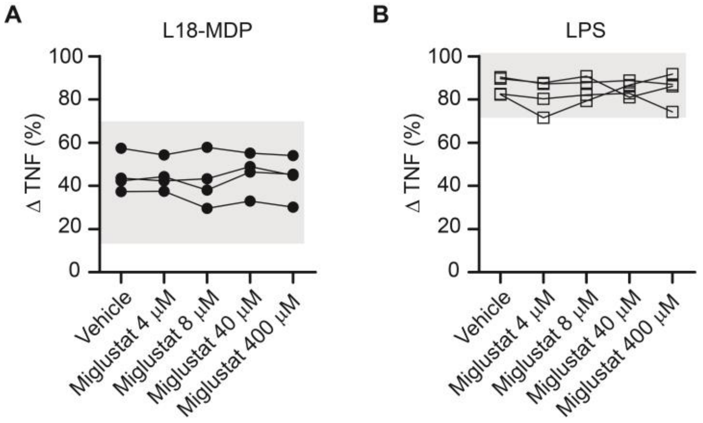

Supplementary figure 6 (related to figure 4). In-vitro miglustat does not affect NOD2 and TLR4 receptor stimulation in monocytes. Analysis of TNF generation in monocytes pre-incubated for 20 hours with vehicle or indicated concentrations of miglustat before stimulation with 200 ng/ml L18-MDP **(A)** or 100 ng/ml LPS **(B)**. Each line represents one healthy individual (n=4). TNF expression was assessed in HLA-DR^+CD14^+ monocytes by intracellular flow cytometry. Δ TNF (%) represents the difference between frequency of TNF-producing monocytes upon stimulation and frequency of TNF-producing monocytes without stimulation. Grey background indicates normal range (mean±2sd) calculated from all measured healthy individuals (n=25).

Supplementary figure 7

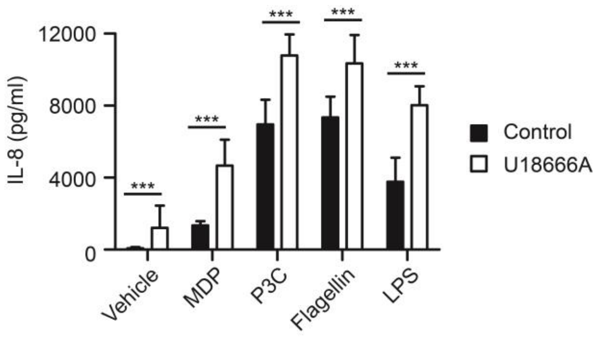

Supplementary figure 7 (related to figure 4). Cytokine release in U18666A-treated macrophages. Macrophages with lysosomal lipid accumulation induced by U18666A secrete more pro-inflammatory IL-8 in response to pattern recognition receptor stimulation. Untreated (control) and U18666A-treated MDM were stimulated with the following ligands: MDP, Pam3CSK4 (P3C), Flagellin or LPS (20 ng/ml). IL-8 in cell culture supernatant was determined six hours after stimulation by ELISA. Data represent mean±sd of independent experiments with three healthy donors performed in triplicates. Statistical significance was determined with Mann-Whitney U test (***P<0.001).

Supplementary figure 8

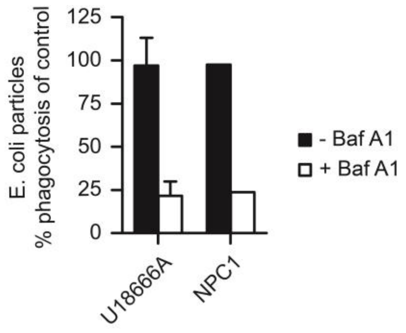

Supplementary figure 8 (related to figure 5). Phagocytosis assay in NPC1 macrophages. Normal phagocytosis of *E. coli* particles by U18666A-treated MDM and NPC1 patient cells. MDM with or without U18666A-induced lysosomal storage phenotype (n=4), primary NPC1 MDM (n=1) and healthy control MDM (n=2) were exposed to *E. coli* bioparticles conjugated to a pH-sensitive dye (pHrodo Red) for 30 minutes, washed and fluorescence measured after 30 minutes time. Fluorescence signal from U18666A-treated MDM was normalized to their respective untreated control MDM and fluorescence signal from NPC1 MDM was normalized to two healthy control MDM measured at the same time. Data represent mean±sd. Treatment of MDM with 50 nM bafilomycin A1 (Baf A1) resulted in a decrease of fluorescence signal.

Supplementary figure 9

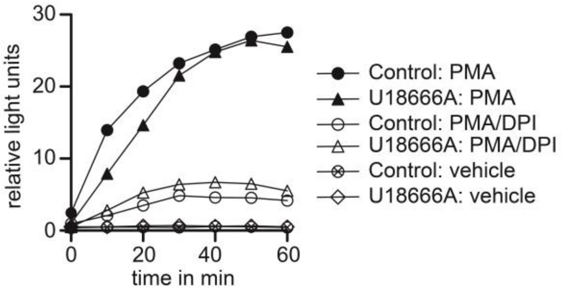

Supplementary figure 9 (related to figure 5). ROS production of U18666A-treated macrophages. Reactive oxygen species (ROS)-production of PMA-activated untreated (control) or U18666A-treated MDM was measured using the chemiluminescence probe L-012. DPI, a NADPH oxidase inhibitor was added as control.

Supplementary figure 10

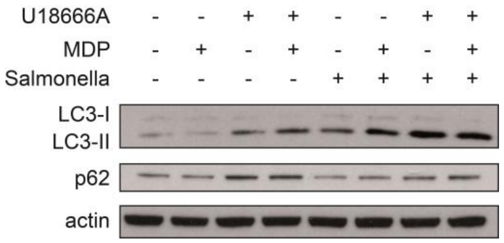

Supplementary figure 10 (related to figure 5). Impaired autophagic flux in U18666A-treated macrophages. High levels of LC3 correlate with increased steady-state amount of autophagosomes and persistence of p62 indicates impaired degradation of internalized cargo contents. U18666A-MDM and untreated MDM were stimulated with MDP and infected with *S. typhimurium* at a MOI of 10 for one hour and then cultured in gentamicin-containing medium for two hours before lysis and immunoblotting for LC3, p62 and actin. Experiments were performed without lysosome/autophagy inhibitors.

Supplementary figure 11

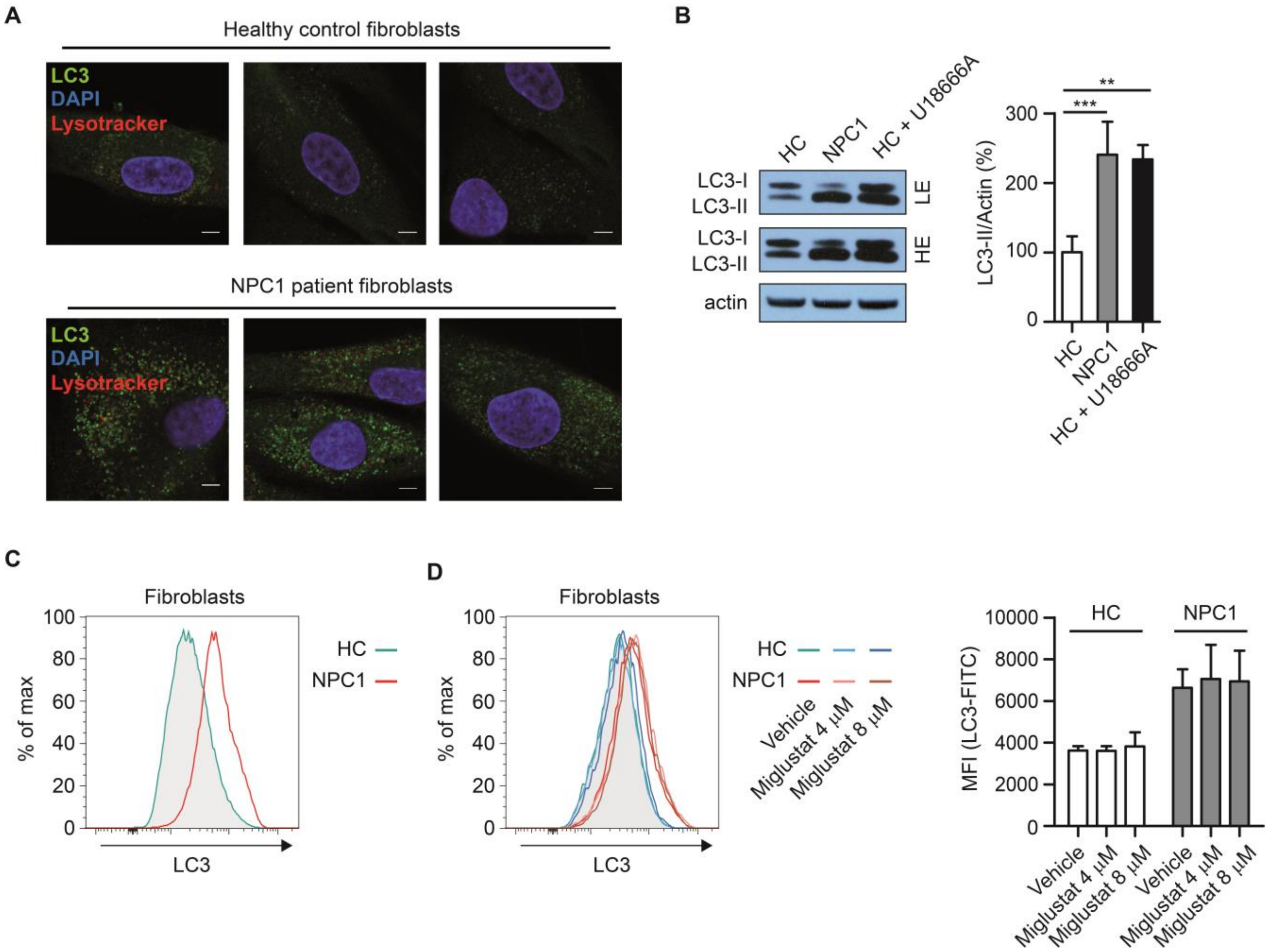

Supplementary figure 11 (related to figure 5). Disturbed baseline autophagic flux in NPC1 patient fibroblasts. Levels of LC3 correlate with numbers of autophagosomes and primary NPC1 fibroblasts exhibit steady state accumulation of autophagosomes. (**A**) Healthy control and NPC1 mutant fibroblasts (compound heterozygote p.I1061T/p.T1036M) were incubated with Lysotracker Red for one hour followed by staining with anti-LC3 antibody and DAPI. Fibroblasts were analysed by confocal microscopy. Scale bar, 5 µm. (**B**) NPC1 and healthy control (HC) fibroblasts were cultured with or without U18666A (2 µg/ml, 48 hours) and cell lysates were immunoblotted for LC3. LC3-levels were quantified relative to actin by densitometry and expressed as percent increase compared to untreated control. Data represent mean±sd of three independent experiments, Mann-Whitney U test (**P<0.01, ***P<0.001). LE, low exposure; HE, high exposure. (**C**) Baseline levels of intracellular LC3 in healthy control (HC) and NPC1 patient fibroblasts were studied with Autophagy Detection Reagent Pack by FACS following the manufacturer's instructions. **(D)** Healthy control (HC) and NPC1 fibroblasts were cultured for five days in complete medium supplemented with miglustat (4 µM or 8 µM) and stained for baseline levels of intracellular LC3. Staining was performed as in (C). Co-culture with miglustat did not decrease elevated levels of autophagosomes in NPC1 fibroblasts. Left: representative flow-cytometric histogram. Right: MFI quantification of LC3-FITC from two independent experiments, mean±sd.

Supplementary figure 12

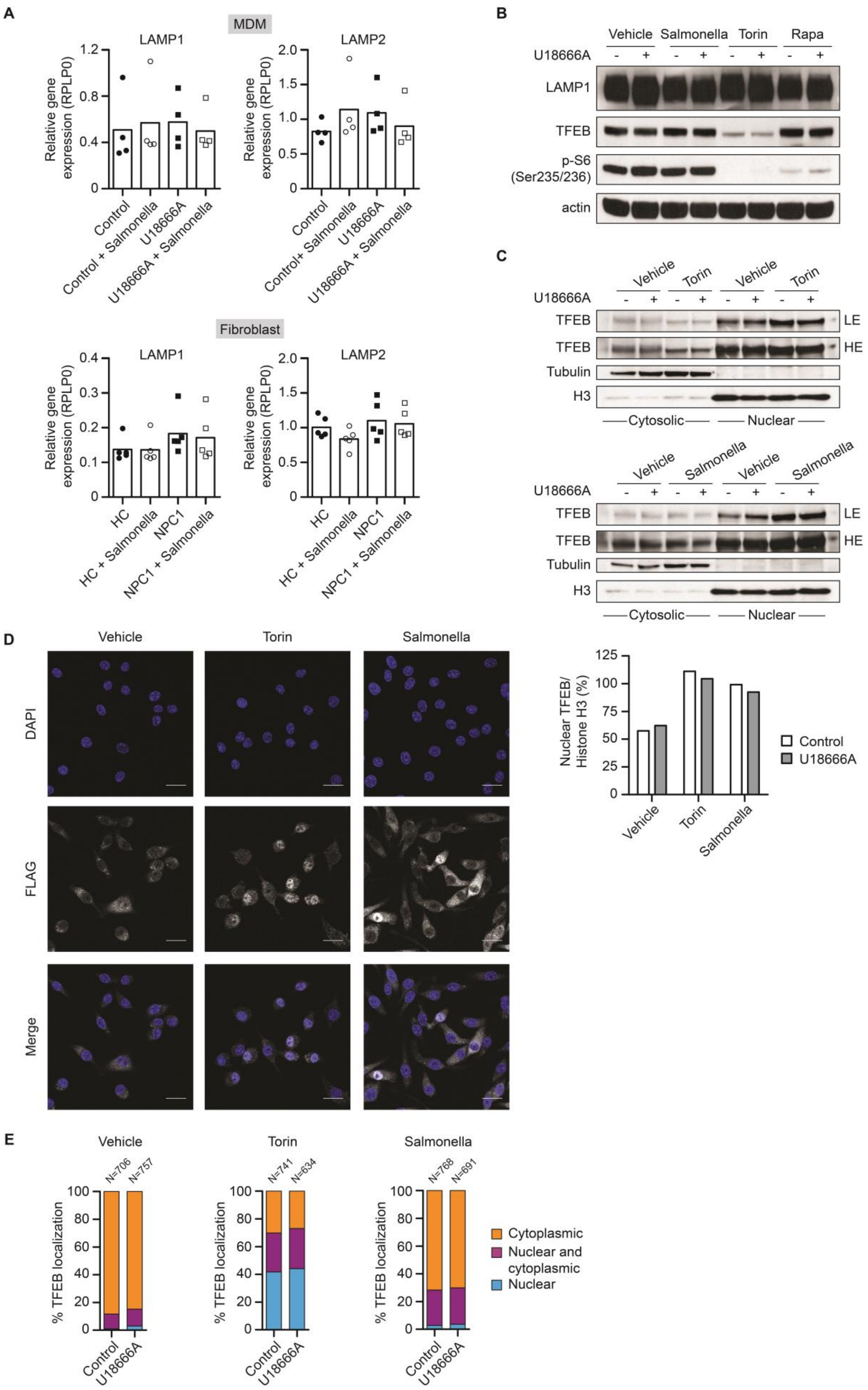

Supplementary figure 12 (related to figure 5). NPC1 lysosomal storage disease does not alter TFEB-target genes LAMP1/2 and shows intact TFEB nuclear translocation upon stimulation with Torin 1 or *S. typhimurium*. (A) Quantitative PCR of TFEB-target genes LAMP1 and LAMP2 in untreated (control) and U18666A-treated MDM generated from four healthy donors (top panel). Expression levels of LAMP1 and LAMP2 in primary human NPC1 (compound heterozygote p.I1061T/p.T1036M) and healthy control (HC) fibroblasts. Macrophages and fibroblasts were infected with *S. typhimurium* at an MOI of 10 for three hours. Results with fibroblasts represent five independent experiments. **(B)** Untreated or U18666A-treated MDM were infected with *S. typhimurium* or stimulated with Torin 1 or rapamycin (both 10 μM) for three hours before cell lysis and immunoblotting for the indicated proteins. Since sample preparation included spinning of cell lysates, results represent the cytosolic fraction of MDM. Immunoblot of endogenous TFEB indicates translocation of the transcription factor to the nucleus following treatment with Torin 1, a strong mTOR inhibitor. Therefore, pharmacological inhibition of mTOR by Torin 1 was used as a positive control for subsequent experiments. Immunoblot of LAMP1 confirms results from pPCR on protein level. Immunoblot of p-S6 (Ser235/236) indicates active signalling via mTOR in untreated and U18666A-treated MDM after treatment with Torin 1 or rapamycin. Results are representative of two independent experiments with different healthy donors. **(C)** Untreated or U18666A-treated MDM were stimulated with Torin 1 (10 μM) or infected with *S. typhimurium* (MOI 10) for three hours before immunoblotting of nuclear and cytosolic fractions for endogenous TFEB. Immunoblotting for tubulin and histone H3 confirms correct subcellular fractionation. Nuclear TFEB-levels were quantified relative to histone H3 by densitometry. Results are representative of two independent experiments with different healthy donors. LE, low exposure; HE, high exposure. **(D)** Representative immunofluorescence staining of TFEB in murine RAW264.7 macrophages stably expressing FLAG-tagged TFEB after treatment with Torin 1 (10 μM, 3 hours) or infection with *S. typhimurium* (MOI 20, 2 hours). Location of TFEB was assessed by microscopy in fixed and permeabilized cells following staining with anti-FLAG (grey) and DAPI (blue). Scale bar, 20 μm. **(E)** Quantification of TFEB localization in TFEB-3xFLAG RAW264.7 cells cultured with our without U18666A and treated as in (D). To improve cell viability of infected RAW264.7 macrophages, induction of lysosomal storage phenotype was performed with 0.5 μg/ml U18666A for 48 hours. Data represent frequency of TFEB localization in >600 cells per condition analysed from three independent experiments. Total numbers of evaluated cells are indicated above bars.

Supplementary figure 13

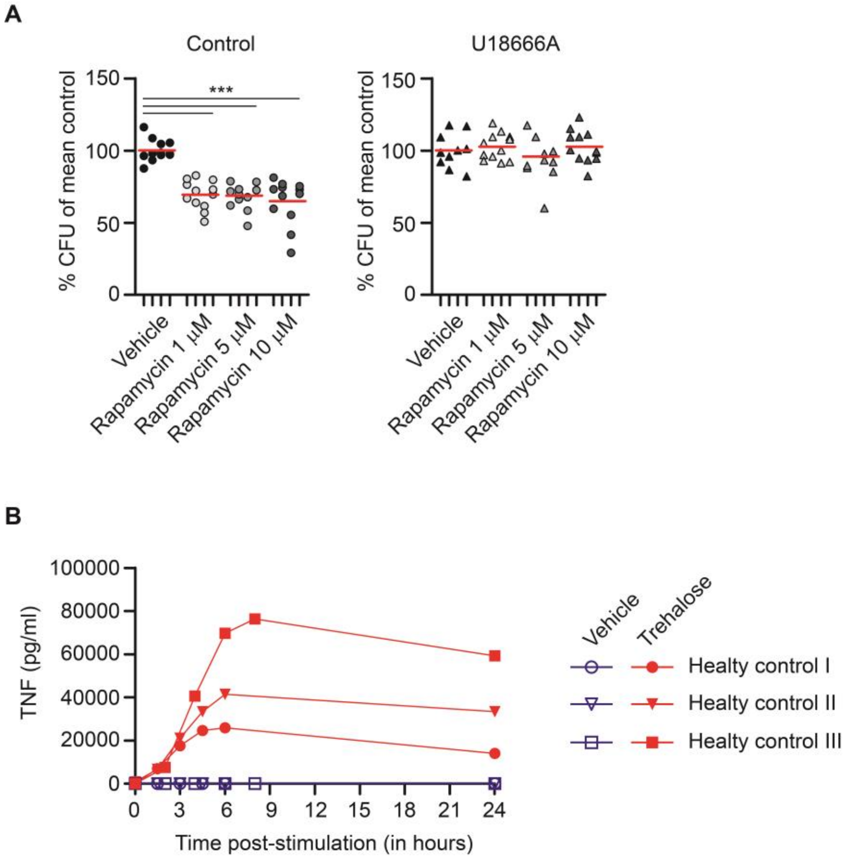

Supplementary figure 13 (related to figure 6). Autophagy inducers rapamycin and trehalose in NPC1. (A) Rapamycin treatment does not overcome defective bacterial handling in U18666A-treated MDM with impaired autophagic flux. Bacterial killing assay was performed with untreated (control) or U18666A-treated MDM pre-incubated with indicated concentrations of rapamycin for three hours before exposure to *S. typhimurium*. Individual results of four healthy donors are shown as indicated by ticks on x-axes. For each donor, conditions were tested in three parallel infection experiments and CFU were normalized to individual CFU without rapamycin. Red bar represents mean. Statistical significance was determined by Mann-Whitney U test (***P<0.001). Concentrations of rapamycin tested in these experiments exceed plasma concentrations found in humans [6]. **(B)** MDM were stimulated with 100 mM trehalose and supernatants stored at indicated time points for analysis by ELISA. Marked induction of pro-inflammatory cytokine TNF by trehalose in human macrophages preclude trehalose as a potential treatment for disturbed bacterial handling in patients. The concentration of trehalose is based on published literature [7].

Supplementary Tables

Supplementary table 1 IBD phenotype in patients with NPC found in the literature

Patient ID	Sex	Age at diagnosis of IBD in years	Diagnosis	Symptoms of IBD / EIM / examination findings	Disease location/ disease behavior *	Treatment for IBD: enteral nutrition, anti-inflammatory/ immunosuppressive drugs	Surgery	Reference
13	F	16	CD	Rectal fistula (persisted despite many treatments)	Perianal disease	ASA		[8] Patient #20
14	M	12	CD	Chronic perianal granulomata, perianal ulcers and abscesses, granulomatous papules and nodular eruptions on the lower abdominal wall	Perianal disease	Systemic and topical CS	Perianal surgery	[9] §

§ The diagnosis is based on liver biopsy and bone marrow aspirate since genetics were not available at that time.

ASA, 5-aminosalicylic acid; CD, Crohn’s disease, CS, corticosteroids; EIM, extraintestinal manifestations; F, female; M, male; * According to Paris classification [1].

Supplementary table 2

IBD patients and NOD2 status				
ID	Gender	Age at time of experiments (years)	IBD type	NOD2 mutation
IBD_1	M	22	CD	R702W/ L1007fsinsC
IBD_2	F	41	CD	R702W/ L1007fsinsC
IBD_3	F	84	CD	R702W/G908R
IBD_4	M	23	CD	R702W/ L1007fsinsC
IBD_5	M	22	CD	G908R/ L1007fsinsC
IBD_6	F	23	CD	R702W/R702W
IBD_7	F	30	CD	R702W/R702W
IBD_8	F	26	CD	L1007fsinsC/ L1007fsinsC
IBD_9	M	23	CD	L1007fsinsC/ L1007fsinsC
IBD_10	F	58	CD	L1007fsinsC/ L1007fsinsC
IBD_11	M	24	CD	L1007fsinsC/ L1007fsinsC
IBD_12	M	50	CD	L1007fsinsC/ L1007fsinsC
IBD_13	F	27	CD	wt/wt
IBD_14	F	25	CD	wt/wt
IBD_15	M	18	CD	wt/wt
IBD_16	M	21	CD	wt/wt
IBD_17	F	37	CD	wt/wt
IBD_18	M	25	CD	wt/wt
IBD_19	M	52	CD	wt/wt
IBD_20	F	40	CD	wt/wt
IBD_21	F	71	CD	wt/wt
IBD_22	M	25	UC	wt/wt
IBD_23	F	43	UC	wt/wt
IBD_24	F	55	UC	wt/wt
IBD_25	F	49	UC	wt/wt
IBD_26	M	25	CD	wt/wt

CD, Crohn’s disease; F, female; IBD, inflammatory bowel disease; M, male; UC, ulcerative colitis; wt, wild-type.

Supplementary table 3

Patients with XIAP mutations				
ID	Gender	Age at time of experiments (years)	IBD	XIAP mutation
XIAP_1.1	M	32	No	G466X
XIAP_1.2	M	36	Yes	G466X
XIAP_1.3	F	43	No	G466X
XIAP_1.4	F	29	No	G466X
XIAP_2	M	3	No	N341YfsX348
XIAP_3	M	7	No	R381X
XIAP_4.1	M	0.33	Yes	K299Lfs307
XIAP_4.2	F	31	No	K299Lfs307

F, female; IBD, inflammatory bowel disease; M, male.

Supplementary References

1. Levine A, Griffiths A, Markowitz J, et al. Pediatric modification of the Montreal classification for inflammatory bowel disease: the Paris classification. *Inflamm Bowel Dis* 2011;**17**(6):1314-21.

2. Ferron M, Settembre C, Shimazu J, et al. A RANKL-PKCbeta-TFEB signaling cascade is necessary for lysosomal biogenesis in osteoclasts. *Genes Dev* 2013;**27**(8):955-69.

3. Ammann S, Elling R, Gyrd-Hansen M, et al. A new functional assay for the diagnosis of X-linked inhibitor of apoptosis (XIAP) deficiency. *Clin Exp Immunol* 2014;**176**(3):394-400.

4. Schindelin J, Arganda-Carreras I, Frise E, et al. Fiji: an open-source platform for biological-image analysis. *Nature methods* 2012;**9**(7):676-82.

5. Damgaard RB, Nachbur U, Yabal M, et al. The ubiquitin ligase XIAP recruits LUBAC for NOD2 signaling in inflammation and innate immunity. *Mol Cell* 2012;**46**(6):746-58.

6. Yatscoff R, LeGatt D, Keenan R, et al. Blood distribution of rapamycin. *Transplantation* 1993;**56**(5):1202-6.

7. Sarkar S, Davies JE, Huang Z, et al. Trehalose, a novel mTOR-independent autophagy enhancer, accelerates the clearance of mutant huntingtin and alpha-synuclein. *The Journal of biological chemistry* 2007;**282**(8):5641-52.

8. Heron B, Valayannopoulos V, Baruteau J, et al. Miglustat therapy in the French cohort of paediatric patients with Niemann-Pick disease type C. *Orphanet J Rare Dis* 2012;**7**:36.

9. Jolliffe DS, Sarkany I. Niemann-Pick type III and Crohn's disease. *J R Soc Med* 1983;**76**(4):307-8.
